# Supplementary material for: Mental healthcare utilisation by patients before and after receiving paliperidone palmitate treatment: mirror image analyses
Source: BMJ Open. 2022 Apr 6;12(4):e051567. doi: 10.1136/bmjopen-2021-051567 (PMC8987753; doi:10.1136/bmjopen-2021-051567)
Supplement: Supplementary data [file bmjopen-2021-051567supp001.pdf]

Supplementary Table 1. Sample characteristics (N=664)

Variables	Number (%) / Mean ± SD
Mean age at index date	42.7 ± 12.8
Gender	
Female	265 (39.9)
Male	399 (60.1)
Most recent employment status at index date	
Unemployed	549 (82.7)
Employed	22 (3.3)
Sick	8 (1.2)
Other	22 (3.3)
Missing	63 (9.5)
Most recent employment status 1 year after index date	
Unemployed	555 (83.6)
Employed	24 (3.6)
Sick	27 (4.1)
Other	36 (5.4)
Missing	22 (3.3)
Current or previous smoker at index date	
No	179 (27.0)
Yes	485 (73.0)
Ethnicity	
White	179 27.0)
Black	399 (60.1)
Asian	36 (5.4)
Other/mixed	50 (7.5)
Marital Status	
Single	521 (78.5)
Married/civil partner/cohabitating	54 (8.1)
Divorced/separated	80 (12.1)
Widowed	9 (1.4)

Most recent Health of the Nation Outcomes Scales (HoNOS) score prior to index date	
Overactive, agitated behaviour	
no problem	315 (48.2)
sub-threshold problem	147 (22.5)
mild but definitely present	103 (15.8)
moderately severe	53 (8.1)
severe to very severe	36 (5.5)
Non-accidental self-injury	
no problem	594 (90.8)
sub-threshold problem	39 (6.0)
mild but definitely present	12 (1.8)
moderately severe	4 (0.6)
severe to very severe	5 (0.8)
Problem-drinking or drug-taking	
no problem	397 (62.0)
sub-threshold problem	82 (12.8)
mild but definitely present	84 (13.1)
moderately severe	57 (8.9)
severe to very severe	20 (3.1)
Cognitive problems	
no problem	354 (54.1)
sub-threshold problem	165 (25.2)
mild but definitely present	90 (13.8)
moderately severe	38 (5.8)
severe to very severe	7 (1.1)
Physical illness or disability problems	
no problem	407 (62.4)
sub-threshold problem	129 (19.8)
mild but definitely present	83 (12.7)
moderately severe	30 (4.6)
severe to very severe	3 (0.5)
Hallucinations and delusions	

no problem	123 (18.9)
sub-threshold problem	128 (19.6)
mild but definitely present	186 (28.5)
moderately severe	145 (22.2)
severe to very severe	70 (10.7)
Depressed mood	
no problem	334 (51.3)
sub-threshold problem	200 (30.7)
mild but definitely present	94 (14.4)
moderately severe	16 (2.5)
severe to very severe	7 (1.1)
Other mental and behavioural problems	
no problem	176 (27.0)
sub-threshold problem	153 (23.5)
mild but definitely present	223 (34.2)
moderately severe	80 (12.3)
severe to very severe	20 (3.1)
Social relationships	
no problem	198 (30.5)
sub-threshold problem	195 (30.1)
mild but definitely present	174 (26.8)
moderately severe	66 (10.2)
severe to very severe	16 (2.5)
Activities of daily living	
no problem	263 (40.5)
sub-threshold problem	183 (28.2)
mild but definitely present	132 (20.3)
moderately severe	58 (8.9)
severe to very severe	14 (2.2)
Standard of living conditions	
no problem	353 (55.9)
sub-threshold problem	137 (21.7)
mild but definitely present	84 (13.3)

moderately severe	35 (5.5)
severe to very severe	23 (3.6)
Occupational and recreational activities	
no problem	235 (37.0)
sub-threshold problem	185 (29.1)
mild but definitely present	150 (23.6)
moderately severe	49 (7.7)
severe to very severe	17 (2.7)
