# Supplementary material for: Mental healthcare utilisation by patients before and after receiving paliperidone palmitate treatment: mirror image analyses
Source: BMJ Open. 2022 Apr 6;12(4):e051567. doi: 10.1136/bmjopen-2021-051567 (PMC8987753; doi:10.1136/bmjopen-2021-051567)
Supplement: Author's manuscript [file bmjopen-2021-051567.draft_revisions.pdf]

BMJ Open is committed to open peer review. As part of this commitment we make the peer review history of every article we publish publicly available.

When an article is published we post the peer reviewers' comments and the authors' responses online. We also post the versions of the paper that were used during peer review. These are the versions that the peer review comments apply to.

The versions of the paper that follow are the versions that were submitted during the peer review process. They are not the versions of record or the final published versions. They should not be cited or distributed as the published version of this manuscript.

BMJ Open is an open access journal and the full, final, typeset and author-corrected version of record of the manuscript is available on our site with no access controls, subscription charges or pay-per-view fees (http://bmjopen.bmj.com).

If you have any questions on BMJ Open's open peer review process please email info.bmjopen@bmj.com

Mental health care utilisation by patients before and after receiving paliperidone palmitate treatment: mirror-image analyses.

Journal:	*BMJ Open*
Manuscript ID	bmjopen-2021-051567
Article Type:	Original research
Date Submitted by the Author:	25-Mar-2021

Complete List of Authors:	Kadra-Scalzo, Giouliana; King's College London<br>Ahn, Deborah; King's College London<br>Bird, Alex; Janssen Pharmaceutical Companies of Johnson and Johnson<br>Broadbent, Matthew; South London and Maudsley Mental Health NHS Trust<br>Chang, Chin-Kuo; King's College London; National Taiwan University, Institute of Epidemiology and Preventive Medicine, College of Public Health<br>Pritchard, Megan; South London and Maudsley Mental Health NHS Trust<br>Shetty, Hitesh; South London and Maudsley Mental Health NHS Trust<br>Taylor, David; South London and Maudsley NHS Foundation Trust<br>Hayes, Richard; King's College London<br>Stewart, Robert; King's College London; South London and Maudsley Mental Health NHS Trust

Keywords:	MENTAL HEALTH, Adult psychiatry < PSYCHIATRY, Schizophrenia & psychotic disorders < PSYCHIATRY

SCHOLARONE™
Manuscripts

BMJ

I, the Submitting Author has the right to grant and does grant on behalf of all authors of the Work (as defined in the below author licence), an exclusive licence and/or a non-exclusive licence for contributions from authors who are: i) UK Crown employees; ii) where BMJ has agreed a CC-BY licence shall apply, and/or iii) in accordance with the terms applicable for US Federal Government officers or employees acting as part of their official duties; on a worldwide, perpetual, irrevocable, royalty-free basis to BMJ Publishing Group Ltd ("BMJ") its licensees and where the relevant Journal is co-owned by BMJ to the co-owners of the Journal, to publish the Work in this journal and any other BMJ products and to exploit all rights, as set out in our licence.

The Submitting Author accepts and understands that any supply made under these terms is made by BMJ to the Submitting Author unless you are acting as an employee on behalf of your employer or a postgraduate student of an affiliated institution which is paying any applicable article publishing charge ("APC") for Open Access articles. Where the Submitting Author wishes to make the Work available on an Open Access basis (and intends to pay the relevant APC), the terms of reuse of such Open Access shall be governed by a Creative Commons licence – details of these licences and which Creative Commons licence will apply to this Work are set out in our licence referred to above.

Other than as permitted in any relevant BMJ Author's Self Archiving Policies, I confirm this Work has not been accepted for publication elsewhere, is not being considered for publication elsewhere and does not duplicate material already published. I confirm all authors consent to publication of this Work and authorise the granting of this licence.

For peer review only

Mental health care utilisation by patients before and after receiving paliperidone palmitate treatment: mirror-image analyses.

Giouliana Kadra-Scalzo[1], Deborah Ahn[1], Alex Bird[2], Matthew Broadbent[3], Chin-Kuo Chang[1,4,5], Megan Pritchard[1,3], Hitesh Shetty[3], David Taylor[3], Richard Hayes*[1] and Robert Stewart*[1,3]

[1] Institute of Psychiatry, Psychology and Neuroscience, King's College London, London, UK

[2] Janssen, UK

[3] South London and Maudsley NHS Foundation Trust, London, UK

[4] Institute of Epidemiology and Preventive Medicine, College of Public Health, National Taiwan University, Taipei City, Taiwan

[5] Global Health Program, College of Public Health, National Taiwan University, Taipei City, Taiwan

*Equal contributions to manuscript

Corresponding author: Dr. Giouliana Kadra-Scalzo, BRC Nucleus, Mapother House, De Crespigny Park, IOPPN, King's College London, London, SE5 8AF, UK; email: giouliana.kadra@kcl.ac.uk;

For peer review only

Abstract

Objectives: To compare mental healthcare use and health care professional (HCP) contacts for patients before and after initiation of paliperidone palmitate.

Setting: The South London and Maudsley NHS Foundation Trust (SLAM) Biomedical Research Centre Clinical Record Interactive Search (CRIS).

Participants: We identified all adults with a diagnosis of schizophrenia (ICD 10: F20.x), who had received paliperidone palmitate prescription for at least 365 days and had at least 1 year of recorded treatment from SLAM, prior to the first recorded receipt of paliperidone palmitate.

Primary and secondary outcome measures: Inpatient and community mental healthcare service use, such as inpatient bed days, number of active days in the service, face-to-face and telephone HCP use in the 12 months before and after paliperidone palmitate initiation.

Results: We identified 664 patients initiated on paliperidone palmitate. Following initiation, inpatient bed days were lower, although patients remained active on the service caseload for longer for both mirror approach 1 [mean difference of inpatient bed days -10.48 (95% CI: (-15.75)- (-5.22)); days active 40.67 (95% CI: 33.39-47.95)] and mirror approach 2 [mean difference of inpatient bed days -23.96 (95% CI: (-30.01)- (-17.92)); mean difference of days active is 40.69 (95% CI: 33.39-47.94)]. The post-initiation period was further characterised by fewer face-to-face and telephone contacts with medical and social work HCPs, and an increased contact with clinical psychologists.

For peer review only - http://bmjopen.bmj.com/site/about/guidelines.xhtml

Conclusions: Our findings indicate a change in the profile of HCP use, consistent with a transition from treatment to rehabilitation.

Strengths and Limitations of this study:

- We captured a large and diverse sample of patients in naturalistic settings, reflective of real-life clinical practice.
- We employed two methods of mirror image analyses, to address the potentially biasing impact of the inpatient episodes within which many initiation events take place.
- The study was observational in nature and therefore the before-after comparisons cannot be assumed to represent causal relationships.
- We were unable to examine the effects of factors, which were not captured by the routine health records.

For peer review only

Background

Treatment discontinuation and covert non-adherence is high amongst patients with serious mental illnesses (1) and current guidelines for schizophrenia recommend that depot medication can be considered (2) to reduce relapse. For patients who have been previously non-adherent, depot antipsychotics have been associated with lower risk of re-hospitalisation (1,3) and relapse (4) in comparison with oral preparations of the same medications and placebos (5). However, there have been significant concerns over extrapyramidal effects (6–8); and the introduction of second-generation (atypical) depot antipsychotics was cited as a potentially significant opportunity for a lower risk of movement disorders (6).

Research into second-generation depot antipsychotics has been relatively sparse. Findings from studies examining risperidone long-acting injection have been mixed with some evidence indicating that risperidone depot is associated with reduced hospital admissions, total in-patient days (9) and improvement in clinical symptoms (10), whereas other studies have suggested that risperidone depot either made no difference (11) or was associated with increased bed stay and healthcare costs (12).

Paliperidone palmitate was introduced in the UK National Health Service in 2011 and is a second-generation antipsychotic depot typically administered once a month intramuscularly. Several studies have investigated the clinical efficacy of paliperidone palmitate in treating schizophrenia symptoms (13,14). Although paliperidone palmitate is associated with higher acquisition cost in comparison to other oral antipsychotics (15), several observational studies have investigated its effectiveness and reported favourable findings in relation to in-patient hospitalization. For example,

For peer review only

Bressington and colleagues (16) reported a reduced number of acute in-patient admissions a year after paliperidone palmitate initiation. Similarly, Taylor and Olofinjana (3) reported a significant decrease in the number and length of hospital admissions per patient per year. Therefore, it appears that although paliperidone palmitate may be associated with a higher initial cost, this may be offset over time through a decrease in rehospitalisation. However, although hospitalisation episodes are strong determinants of healthcare costs, it is also important to understand health care professional (HCP) contacts, which reflect intensity of input required in outpatient settings. To the authors' knowledge, there has been no research to date that has examined secondary mental health care use in this level of detail. Consequently the aim of this study was to describe inpatient and community mental healthcare service use, including HCP contacts, of schizophrenia patients being treated with paliperidone palmitate, specifically comparing the frequency and duration of HCP contacts before and after initiation of paliperidone palmitate therapy, using mirror-image methodologies.

For peer review only

For peer review only - http://bmjopen.bmj.com/site/about/guidelines.xhtml

Methods

The data for this study were sourced from the South London and Maudsley NHS Foundation Trust (SLAM) Biomedical Research Centre Clinical Record Interactive Search (CRIS). CRIS has been previously described in detail (17,18); briefly this is a data resource sourced from the electronic mental health records of SLAM, which provides comprehensive mental health services to a geographic catchment of 1.36 million residents in four boroughs of south London. All records are de-identified by CRIS and made available for research use under the governance framework (19). CRIS data have been substantially enhanced through the application of natural language processing to text fields (18).

We identified all adults with a diagnosis of schizophrenia (ICD 10: F20.x), who had received paliperidone palmitate prescription for at least 365 days and had at least 1 year of recorded treatment from SLAM, continuous or not, prior to the first recorded receipt of paliperidone palmitate. The first recorded receipt of paliperidone palmitate was defined as the 'index date' for definitions of outcomes and covariates and all patients were followed-up for one year from their index date.

A range of covariates were identified for cohort characterisation. These included age on the index date, gender, and recorded ethnicity grouped into the following categories: white, black, Asian or mixed/other. Marital status was derived from a clinician-completed compulsory structured field in the source record, and was grouped into the following categories: single; married / civil partnership / cohabiting; divorced / separated; widowed. Employment status was derived from compulsory structured fields and referred to the most recent recording on or prior to the index date. Smoking status was derived from a combination of structured fields in the

For peer review only

record and a natural language processing algorithm ascertain text inferring smoking status (17); current or previous smoking in the record was ascertained prior to the index date. The Health of the Nation Outcome Scales (HoNOS) instrument is routinely used in SLAM services for monitoring purposes, and individual scales from this instrument were extracted as 5-point scores (no problem, minimal, mild, moderate and severe problem). HCP contacts were quantified from structured meta-data for SLAM case note entries, ascertaining contacts on the basis of an 'attended' encounter recorded as either face-to-face or by phone. The profession of the person posting each entry was ascertained from a SLAM Human Resources database of named staff mapped to professional groups. Within the intervals of interest, durations of time were also calculated that each individual spent as an inpatient, and 'active' to SLAM (i.e. receiving assessment/care and not discharged).

Ethics approval

CRIS is approved for secondary analysis by the Oxfordshire Research Ethics Committee C (reference 18/SC/0372).

Patient and public involvement

The project was reviewed and approved by the CRIS Oversight Committee. All CRIS related research projects are considered and approved by a patient-led Oversight Committee, reporting to the Caldicott Guardian. The committee considers the appropriateness of the research proposals and adjudicates on risks of de-anonymisation at the analysis planning stage.

Statistical analyses

For peer review only

We used STATA 13 for all statistical analyses. Sample characteristics were summarised for the total cohort. We compared HCP use between the 12 months before and after initiation, choosing this time period *a priori* due to the risk of selection (attrition) bias for longer post-initiation periods. Mean numbers of HCP contacts were compared between the two periods of interest within individuals in the sample using Wilcoxon signed-rank tests. The median and interquartile range (IQR) are also provided for all HCP contacts. Figure 1 describes all methodologies adopted to investigate the data: a) the unadjusted analysis reflects the unmodified pre-/post-initiation comparisons of HCP contacts; b) in mirror approach 1, for service users who were inpatients at the time of their paliperidone palmitate initiation, the post-initiation inpatient period was excluded as well as an equivalent period pre-initiation to minimise potential bias from extra inpatient days required for paliperidone palmitate stabilisation, whilst ensuring that comparisons were made between periods of identical duration. For all outpatients no time was excluded; c) in mirror approach 2, for service users who were inpatients at the time of their paliperidone palmitate initiation, the period between paliperidone palmitate initiation and in-patient discharge was excluded, as this is the period when the new medication is stabilising. In the latter mirror image approach, the days between hospitalisation and paliperidone palmitate initiation are attributed to the 'before' period, following the assumption that the hospitalisation period is due to the treatment failure of the previous antipsychotic (3). For all outpatients no time was excluded.

For peer review only - http://bmjopen.bmj.com/site/about/guidelines.xhtml

Results

We identified 664 patients with schizophrenia who met the inclusion criteria; 375 (56.5) had paliperidone palmitate initiated in inpatient settings and 289 (43.5) had paliperidone palmitate initiated in outpatient settings. Of people with schizophrenia receiving paliperidone palmitate for at least a year, 39.5 % (n=262) were documented as receiving it for at least 3 years. Table 1 describes the socio-demographic, socioeconomic and clinical symptoms characteristics of the cohort. The mean age was 43 years, 60% of the sample were male, 60% of black ethnicity, close to 80% were single, and over 80% were unemployed. Close to three quarters were recorded as being current smokers. From most recent HoNOS scores, significant overactive/agitated behaviour was present in around 30% of the sample at initiation, around 25% had problematic alcohol or substance use, around 20% had significant cognitive problems, over 60% had significant hallucinations and/or delusions; however, the prevalence of depressive and self-harm problems were both below 20%. Significant difficulties in social relationships were present in approximately 40%, impaired activities of daily living in around 30%, significant problems with living conditions in over 20%, and impaired occupational/recreational activity in around 35%.

Table 2 summarises the unmodified analyses comparing mental healthcare and HCP use before and after paliperidone palmitate initiation. Overall, face-to-face contacts were significantly lower after initiation for nurses [mean difference -2.38 (95%CI: (-3.69)- (-1.07)], social workers [mean difference -0.64 (95%CI: (-1.08)- (-0.19))], medical professionals [mean difference -0.58 (95%CI:(-0.81)- (-0.34))], and unspecified staff members [mean difference -0.42 (95%CI: (-0.76)- (-0.07))].

For peer review only - http://bmjopen.bmj.com/site/about/guidelines.xhtml

However, contact with clinical psychologists was increased in the post-initiation period [mean difference 0.27 (95%CI: (-0.01)-0.56)]. Contact with occupational therapists in the post-initiation period was also higher but difference did not reach statistical significance. Telephone HCP contacts showed similar differences. Inpatient bed days [mean difference 13.93 (95%CI: 7.31- 20.54)] and days 'active' to SLAM [mean difference 40.67 (95%CI: 33.39- 47.95)] were significantly higher during the post-initiation period.

Table 3 describes results for the first mirror image analysis, where paliperidone palmitate post-initiation inpatient period was excluded as well as an equivalent period pre-initiation. The post-initiation period was characterised with significantly lower face-to-face contact with social workers [mean difference -0.06 (95%CI: (-0.49)- 0.37)], medical professionals [mean difference -0.31 (95% CI: (-0.54)- (-0.08))] and unspecified professionals [mean difference -0.41 (95% CI: (-0.76)- (-0.07))]. The post-initiation period was also characterised with significantly lower telephone contact with social workers [mean difference -0.42 (95% CI: (-0.76)- (-0.07))], medical professionals [mean difference -0.08 (95% CI: (-0.13)- (-0.03))] and unspecified professionals [mean difference -0.17 (95% CI: (-0.29)- (-0.06))]. However, face-to-face and telephone contact with clinical psychologists [face-to-face mean difference 0.27 (95% CI: (-0.01)- 0.56); telephone mean difference 0.05 (95% CI: (-0.03)- 0.13)] was significantly higher than during the pre-initiation period. In the post-initiation period, telephone contact with occupational therapist was also significantly higher [mean difference 0.27 (95% CI: 0.05- 0.50)]. The post-initiation period was also characterised in this model by fewer inpatient bed days [mean

For peer review only - http://bmjopen.bmj.com/site/about/guidelines.xhtml

difference -10.48 (95% CI: (-15.74)- (-5.22))] and an increase in days active to SLAM services [mean difference 40.67 (95% CI: 33.39-47.94)].

Table 4 illustrates the analysis for the second mirror image approach, where the period between paliperidone palmitate initiation and in-patient discharge was excluded. Overall face-to-face contacts were lower after paliperidone palmitate initiation – this was statistically significant for nurses [mean difference -2.38 (95% CI: (-3.69)- (-1.07)], social workers [mean difference -0.64 (95% CI: (-1.08)- (-0.19))], medical professionals [mean difference -0.58 (95% CI: (-0.81)- (-0.34))], and unspecified staff members [mean difference -0.42 (95% CI: (-0.76)- (-0.07))]. In addition, telephone contacts were significantly lower after paliperidone palmitate initiation for nurses [mean difference -1.17 (95% CI: (-1.73)- (-0.60))], social workers [mean difference -0.97 (95% CI: (-1.35)- (-0.59))], medical staff [mean difference -0.11 (95% CI: (-0.17)- (-0.05))] and unspecified staff members [mean difference -0.17 (95% CI: (-0.29)- (-0.06))]. We further observed an increased face-to-face and telephone contact with clinical psychologists [mean difference -0.27 (95% CI: (-0.02)- 0.56), and mean difference 0.05 (95% CI: (-0.03)- 0.14), respectively]. There was also a significant decrease in inpatient bed days post-initiation [mean difference -23.96 (95% CI: (-30.01)- (-17.92))], and higher number of days 'active' to SLAM [mean difference 40.69 (95% CI: 33.39- 47.94)].

For peer review only - http://bmjopen.bmj.com/site/about/guidelines.xhtml

Discussion

To our knowledge, this is the first study to investigate HCP use in secondary mental healthcare associated with paliperidone palmitate initiation. In a large mental healthcare database, we found that the initiation of paliperidone palmitate was associated with significantly decreased face-to-face and telephone contact with medical professionals, social workers and unspecified HCP and increased contact with therapeutic services such as clinical psychologists. This, in addition to the higher proportion of time spent 'active' in SLAM after initiation, and a decrease in days spend as an inpatient is consistent with a shift from treatment and risk management to rehabilitation. The results were largely consistent irrespective of the mirror image approach that was employed to take into account the potentially biasing effects of inpatient care at the time of treatment initiation, although reduction in nursing input post-initiation was only evident in the unadjusted analysis and second mirror image approach (but not in mirror image approach one).

Considering hospitalisation, our results are consistent with previous research findings that paliperidone palmitate initiation is associated with a reduction in inpatient bed days after the two mirror approaches were applied (3,16); we were able to replicate this in a larger cohort of patients initiated on paliperidone palmitate than previously reported, as well as using within-individual comparisons that will have removed the influence of between-individual differences as confounding factors. Taylor and Olofinjana (3) have argued that there are several possible mechanisms that may explain this assumed effect of paliperidone palmitate, including the ability to give loading doses and higher equivalent doses compared to risperidone depot.

For peer review only - http://bmjopen.bmj.com/site/about/guidelines.xhtml

However, ultimately a formal randomised controlled trial would be required to assess this.

The post-initiation reduction in contact with medical and social work staff in both models, and reduction in nursing staff contact in the second model, may reflect similar improvements in mental state and is consistent with a wider benefit of treatment initiation than is indicated by inpatient care comparisons. Furthermore, the increased contact with clinical psychology staff does not support an overall reduction in HCP contact but instead an altered profile of contact from staff members responsible for treatment compared to those responsible for rehabilitation. It should be borne in mind that no control condition was included in the design – i.e. the apparent HCP changes might have been observed regardless of the date chosen to divide comparison periods, and/or samples receiving inpatient care may naturally have higher medical and social work involvement prior to an admission episode. Furthermore, if changes in HCP contacts represent an effect of the intervention rather than inpatient care, it was not possible to subdivide the sample in terms of conditions prior to the event (i.e. the precise medication change being instituted).

This study had several strengths. We examined a large and diverse sample of patients in naturalistic settings. Therefore, our sample should be maximally reflective of health care professional contact in real-world clinical settings. The use of mental healthcare data was justified because very few paliperidone palmitate initiations would be expected outside specialist services. Furthermore, we had sufficient statistical power to characterise the sample according to a diverse number of patient characteristics, and the within-patient comparisons removes the effect of between-patient differences as confounding factors. Finally, in addition to the unadjusted

For peer review only - http://bmjopen.bmj.com/site/about/guidelines.xhtml

analysis, we employed two methods of mirror image analyses, to address the potentially biasing impact of the inpatient episodes within which many initiation events took place.

On the other hand, several limitations need to be borne in mind. The study was observational in nature and therefore the before-after comparisons cannot be assumed to represent causal relationships (i.e. a treatment effect). In addition, the data were limited by availability from a routine health record and therefore we were unable to examine factors underlying changes in HCP contacts, such as clinical symptoms before and after paliperidone palmitate and prior medication history. In addition, selecting patients who have taken paliperidone palmitate for a year, are likely to represent a group of patients who have good outcomes. Mace and colleagues (20) have indicated that patients with longer duration of illness, who have been initiated on long-acting injectables such as haloperidol deconate, for reasons other than non-adherence, are more likely to remain on their treatment. Furthermore, we did not examine the formulation of paliperidone palmitate (e.g. monthly; three-monthly), however at the time the study was conducted we believe the monthly formulation was mostly in use. In addition, a longer follow-up period would have allowed us to investigate longer-term effect of paliperidone palmitate and therefore should be considered by further research. It is also important to consider that follow-up began at the point paliperidone palmitate was initiated as opposed to hospital discharge. Furthermore, we did not include a control group of another depot or oral antipsychotic, therefore we cannot infer that changes associated with paliperidone palmitate initiation would not have been observed with other interventions.

For peer review only - http://bmjopen.bmj.com/site/about/guidelines.xhtml

Overall, our findings suggest that paliperidone palmitate initiation is associated with a change in healthcare professional contact which could indicate change in patients' presentation. However, further research is needed that focuses on examining changes in mental health symptoms from the before to after period to support this hypothesis. Finally, we did not seek to quantify the economic impact of changes in health service use, and further research would be needed to determine whether this translates into lower overall service costs.

For peer review only - http://bmjopen.bmj.com/site/about/guidelines.xhtml

Authors' contribution:

GKS drafted the manuscript and together with DA conducted the analyses. MB, MP and HS put together the data extraction plan and extracted the data. AB, CK-C, DT, RH and RS contributed to the conceptualization and designing the study. All authors critically revised the manuscript and have approved the final version.

Competing interests: RDH, C-KC, HS and RS have received research funding from Roche, Pfizer, Janssen and Lundbeck. GKS has received funding from Janssen and Lundbeck. RS has also received research funding from Takeda. AB is an employee of Janssen, UK. DT has received investigator-initiated research grants from Astra-Zeneca, Eli Lilly, Janssen, Lundbeck, Otsuka, Servier, Sunovion.

Funding: The study described here was funded by Janssen UK. This work was supported by the Clinical Record Interactive Search (CRIS) platform funded and developed by the National Institute for Health Research (NIHR) Biomedical Research Centre at South London and Maudsley NHS Foundation Trust and King's College London, and a joint infrastructure grant from Guy's and St Thomas' Charity and the Maudsley Charity (grant number BRC-2011-10035). RDH, RS, HS, C-KC, RDH and GKS receive salary support from the National Institute for Health Research (NIHR) Biomedical Research Centre at South London and Maudsley NHS Foundation Trust and King's College London. DT is a pharmacist and clinical academic at the South London and Maudsley Hospital NHS Trust and King's College London. The views expressed are those of the authors and not necessarily those of the NHS, the NIHR or the Department of Health.

For peer review only - http://bmjopen.bmj.com/site/about/guidelines.xhtml

Data availability: The data that support the findings of this study are available on request from the corresponding author. The data are not publicly available due to the Information Governance framework and REC approval in place concerning CRIS data use.

For peer review only

For peer review only - http://bmjopen.bmj.com/site/about/guidelines.xhtml

References

1. Tiihonen J, Haukka J, Taylor M, Haddad PM, Patel MX, Korhonen P. A nationwide cohort study of oral and depot antipsychotics after first hospitalization for schizophrenia. Am J Psychiatry. 2011;168(6):603–9.

2. NICE, NCCMH. Schizophrenia. The nice guideline on core interventions in the treatment and management of schizophrenia in adults in primary and secondary care. NICE; 2013.

3. Taylor D, Olofinjana O. Long-acting paliperidone palmitate - Interim results of an observational study of its effect on hospitalization. Int Clin Psychopharmacol. 2014;29(4):229–34.

4. Leucht C, Heres S, Kane JM, Kissling W, Davis JM, Leucht S. Oral versus depot antipsychotic drugs for schizophrenia-A critical systematic review and meta-analysis of randomised long-term trials. Schizophr Res. 2011;127(1–3):83–92.

5. Kozma CM, Slaton T, Dirani R, Fastenau J, Gopal S, Hough D. Changes in schizophrenia-related hospitalization and ER use among patients receiving paliperidone palmitate: results from a clinical trial with a 52-week open-label extension (OLE). Curr Med Res Opin. 2011;27(8):1603–11.

6. Leucht S, Pitschel-Walz G, Abraham D, Kissling W. Efficacy and extrapyramidal side-effects of the new antipsychotics olanzapine, quetiapine, risperidone, and sertindole compared to conventional antipsychotics and placebo. A meta-analysis of randomized controlled trials. Schizophr Res. 1999;35(1):51–68.

7. Barnes TRE, Cursori DA. Long Term Depot Antipsychotics: A Risk-Benefit Assessment. Drug Saf. 1994;10(6):464–79.

8. Novick D, Haro JM, Perrin E, Suarez D, Texeira JM. Tolerability of outpatient antipsychotic treatment: 36-month results from the European Schizophrenia Outpatient Health Outcomes (SOHO) study. Eur Neuropsychopharmacol. 2009;19(8):542–50.

9. Niaz OS, Haddad PM. Thirty-five months experience of risperidonelong-acting injection in a UK psychiatric service including a mirror-image analysis of in-patient care. Acta Psychiatr Scand. 2007;116(1):36–46.

10. Taylor DM, Young C, Patel MX. Prospective 6-month follow-up of patients prescribed risperidone long-acting injection: Factors predicting favourable outcome. Int J Neuropsychopharmacol. 2006;9(6):685–94.

11. Taylor D, Fischetti C, Sparshatt A, Thomas A, Bishara D, Cornelius V. Risperidone long-acting injection: A 6-year mirror-image study of healthcare resource use. Acta Psychiatr Scand. 2009;120(2):97–101.

12. Young CL, Taylor DM. Health resource utilization associated with switching to risperidone long-acting injection. Acta Psychiatr Scand. 2006;114(1):14–20.

For peer review only

For peer review only - http://bmjopen.bmj.com/site/about/guidelines.xhtml

13. Hough D, Gopal S, Vijapurkar U, Lim P, Morozova M, Eerdekens M. Paliperidone palmitate maintenance treatment in delaying the time-to-relapse in patients with schizophrenia: A randomized, double-blind, placebo-controlled study. Schizophr Res. 2010;116(2–3):107–17.

14. Gopal S, Vijapurkar U, Lim P, Morozova M, Eerdekens M, Hough D. A 52-week open-label study of the safety and tolerability of paliperidone palmitate in patients with schizophrenia. J Psychopharmacol. 2011;25(5):685–97.

15. Lefebvre P, Muser E, Duh MS, DerSarkissian M, Faust E, Kageleiry A, et al. Impact of paliperidone palmitate versus oral atypical antipsychotics on healthcare resource use and costs in veterans with schizophrenia. Amcp 2015. 2015;4:579–92.

16. Bressington D, Stock J, Hulbert S, MacInnes D. A retrospective observational study of the effectiveness of paliperidone palmitate on acute inpatient hospitalization rates. Int Clin Psychopharmacol. 2015;30(4):230–6.

17. Perera G, Broadbent M, Callard F, Chang C-K, Downs J, Dutta R, et al. Cohort profile of the South London and Maudsley NHS Foundation Trust Biomedical Research Centre (SLaM BRC) Case Register: Current status and recent enhancement of an Electronic Mental Health Record-derived data resource. BMJ Open. 2016;6(3).

18. Stewart R, Soremekun M, Perera G, Broadbent M, Callard F, Denis M, et al. The South London and Maudsley NHS Foundation Trust Biomedical Research Centre (SLAM BRC) case register: development and descriptive data. BMC Psychiatry. 2009;9(1):51.

19. Fernandes AC, Cloete D, Broadbent MTM, Hayes RD, Chang C-K, Jackson RG, et al. Development and evaluation of a de-identification procedure for a case register sourced from mental health electronic records. BMC Med Inform Decis Mak. 2013;13(1):71.

20. Mace S, Dzahini O, O'Hagan M, Taylor D. Haloperidol decanoate long-acting injection (HDLAI): Results of a 1-year mirror-image study. Ther Adv Psychopharmacol. 2018;8(9):241–9.

For peer review only

For peer review only - http://bmjopen.bmj.com/site/about/guidelines.xhtml

Table 1. Sample characteristics (N=664)

Variables	Number (%) / Mean ± SD
Mean age at index date	42.7 ± 12.8
Gender	
Female	265 (39.9)
Male	399 (60.1)
Most recent employment status at index date	
Unemployed	549 (82.7)
Employed	22 (3.3)
Sick	8 (1.2)
Other	22 (3.3)
Missing	63 (9.5)
Most recent employment status 1 year after index date	
Unemployed	555 (83.6)
Employed	24 (3.6)
Sick	27 (4.1)
Other	36 (5.4)
Missing	22 (3.3)
Current or previous smoker at index date	
No	179 (27.0)
Yes	485 (73.0)
Ethnicity	
White	179 27.0)
Black	399 (60.1)
Asian	36 (5.4)
Other/mixed	50 (7.5)
Marital Status	
Single	521 (78.5)
Married/civil partner/cohabitating	54 (8.1)
Divorced/separated	80 (12.1)

For peer review only - http://bmjopen.bmj.com/site/about/guidelines.xhtml

For peer review only

Widowed	9 (1.4)
Most recent Health of the Nation Outcomes Scales (HoNOS) score prior to index date	
Overactive, agitated behaviour	
no problem	315 (48.2)
sub-threshold problem	147 (22.5)
mild but definitely present	103 (15.8)
moderately severe	53 (8.1)
severe to very severe	36 (5.5)
Non-accidental self-injury	
no problem	594 (90.8)
sub-threshold problem	39 (6.0)
mild but definitely present	12 (1.8)
moderately severe	4 (0.6)
severe to very severe	5 (0.8)
Problem-drinking or drug-taking	
no problem	397 (62.0)
sub-threshold problem	82 (12.8)
mild but definitely present	84 (13.1)
moderately severe	57 (8.9)
severe to very severe	20 (3.1)
Cognitive problems	
no problem	354 (54.1)
sub-threshold problem	165 (25.2)
mild but definitely present	90 (13.8)
moderately severe	38 (5.8)
severe to very severe	7 (1.1)
Physical illness or disability problems	
no problem	407 (62.4)
sub-threshold problem	129 (19.8)
mild but definitely present	83 (12.7)
moderately severe	30 (4.6)
severe to very severe	3 (0.5)

For peer review only

Hallucinations and delusions	
no problem	123 (18.9)
sub-threshold problem	128 (19.6)
mild but definitely present	186 (28.5)
moderately severe	145 (22.2)
severe to very severe	70 (10.7)
Depressed mood	
no problem	334 (51.3)
sub-threshold problem	200 (30.7)
mild but definitely present	94 (14.4)
moderately severe	16 (2.5)
severe to very severe	7 (1.1)
Other mental and behavioural problems	
no problem	176 (27.0)
sub-threshold problem	153 (23.5)
mild but definitely present	223 (34.2)
moderately severe	80 (12.3)
severe to very severe	20 (3.1)
Social relationships	
no problem	198 (30.5)
sub-threshold problem	195 (30.1)
mild but definitely present	174 (26.8)
moderately severe	66 (10.2)
severe to very severe	16 (2.5)
Activities of daily living	
no problem	263 (40.5)
sub-threshold problem	183 (28.2)
mild but definitely present	132 (20.3)
moderately severe	58 (8.9)
severe to very severe	14 (2.2)
Standard of living conditions	
no problem	353 (55.9)
sub-threshold problem	137 (21.7)

For peer review only

mild but definitely present	84 (13.3)
moderately severe	35 (5.5)
severe to very severe	23 (3.6)
Occupational and recreational activities	
no problem	235 (37.0)
sub-threshold problem	185 (29.1)
mild but definitely present	150 (23.6)
moderately severe	49 (7.7)
severe to very severe	17 (2.7)

For peer review only

For peer review only - http://bmjopen.bmj.com/site/about/guidelines.xhtml

Table 2. Frequencies of health care professionals contacts during the 12 month periods before and after the index date – unmodified analysis of the total time periods

Type of contact	Mean ± SD number of contacts		Median (Interquartile range)		P-value*
	Before paliperidone palmitate initiation	After paliperidone palmitate initiation	Before paliperidone palmitate initiation	After paliperidone palmitate initiation	
Face-to-face contact					
Total	29.70 ± 24.90	25.8 ± 20.40	26 (24)	22 (17)	<0.001
Nurse	17.60 ± 16.90	15.3 ± 12.74	15 (22)	13 (11)	<0.001
Social worker	4.00 ± 5.90	3.32 ± 6.12	2 (5)	1 (3)	<0.001
Medical	1.74 ± 3.00	1.16 ± 2.13	1 (2)	0 (1)	<0.001
Consultant	1.76 ± 2.57	1.53 ± 2.20	1 (2)	1 (2)	0.07
Occupational therapist	0.70 ± 2.51	0.94 ± 3.30	0 (0)	0 (0)	0.41
Administrative	1.07 ± 2.90	0.97 ± 3.10	0 (1)	0 (1)	0.09
Unspecified	1.33 ± 4.66	0.91 ± 3.11	0 (1)	0 (0)	<0.001
Health Care Assistant	0.83 ± 6.23	0.76 ± 3.69	0 (0)	0 (0)	0.34
Clinical psychologist	0.58 ± 2.61	0.85 ± 3.63	0 (0)	0 (0)	0.04

For peer review only - http://bmjopen.bmj.com/site/about/guidelines.xhtml

Other therapist	0.08 ± 0.77	0.08 ± 0.96	0 (0)	0 (0)	0.44
Phone contact					
Total	11.03 ± 10.45	8.74 ± 8.74	8 (13.5)	6 (11)	<0.001
Nurse	6.06 ± 7.41	4.89 ± 5.86	3 (8)	3 (6)	<0.001
Social worker	2.98 ± 5.73	2.01 ± 4.65	1 (3)	0 (2)	<0.001
Medic	0.21 ± 0.82	0.10 ± 0.40	0 (0)	0 (0)	0.001
Consultant	0.18 ±0.74	0.13 ± 0.48	0 (0)	0 (0)	0.87
Occupational therapist	0.52 ± 2.22	0.71 ± 2.80	0 (0)	0 (0)	0.19
Administrative	0.39 ± 1.20	0.34 ± 1.12	0 (0)	0 (0)	0.20
Unspecified	0.39 ± 1.43	0.22 ± 0.80	0 (0)	0 (0)	0.001
Health Care Assistant	0.10 ± 0.60	0.08 ± 0.58	0 (0)	0 (0)	0.13
Clinical psychologist	0.19 ± 0.92	0.24 ± 1.06	0(0)	0 (0)	0.03
Other therapist	0.02 ± 0.21	0.01 ± 0.18	0 (0)	0(0)	0.21
Inpatient bed days	45.06 ± 64.00	58.99 ± 87.84	20 (61)	23.5 (79.5)	0.01
Days active†	322.81 ± 95.63	363.28 ± 13.54	365 (0)	365 (0)	<0.001

* Testing the individual differences between the two periods using Wilcoxon signed-rank test
† Days active are calculated from the total number of days on the SLAM caseload during the relevant period – i.e. excluding days between SLAM referrals

For peer review only - http://bmjopen.bmj.com/site/about/guidelines.xhtml

Table 3. Frequencies of health care professionals contacts during the 12 month periods before and after the index date – 'mirror' analysis excluding the influence of the inpatient period around paliperidone palmitate initiation (mirror approach 1)

Type of contact	Mean ± SD number of contacts		Median (Interquartile range)		P-value*
	Before paliperidone palmitate initiation	After paliperidone palmitate initiation	Before paliperidone palmitate initiation	After paliperidone palmitate initiation	
Face-to-face contact					
Total	26.87 ± 24.92	25.79 ± 20.37	23 (24)	22 (17)	0.29
Nurse	16.25 ± 16.62	15.25 ± 12.74	13 (21)	13 (11)	0.40
Social worker	3.39 ± 5.58	3.33 ± 6.12	1 (4)	1 (3)	0.03
Medical staff	1.47 ± 2.86	1.16 ± 2.13	0 (2)	0 (1)	<0.01
Consultant	1.76 ± 2.57	1.53 ± 2.20	1 (2)	1 (2)	0.07
Occupational therapist	0.62 ± 2.34	0.94 ± 3.30	0 (0)	0(0)	0.09
Administrative	0.97 ± 2.81	0.97 ± 3.09	0 (1)	0 (1)	0.59
Unspecified	1.33 ± 4.66	0.91 ± 3.11	0 (1)	0 (0)	<0.001
Health Care Assistant	0.77 ± 6.14	0.76 ± 3.69	0(0)	0(0)	0.07
Clinical psychologist	0.58 ± 2.61	0.85 ± 3.63	0(0)	0(0)	0.04

For peer review only

Other therapist	0.07 ± 0.77	0.08 ± 0.96	0(0)	0(0)	0.44
Phone contact					
Total	9.38 ± 9.57	8.74 ± 8.74	6.5 (12)	6 (11)	0.03
Nurse	5.28 ± 6.71	4.89 ± 5.86	3 (8)	3 (6)	0.29
Social worker	2.43 ± 5.18	2.01 ± 4.65	1 (2)	0 (2)	<0.001
Medical staff	0.18 ± 0.75	0.10 ± 0.40	0 (0)	0 (0)	0.02
Consultant	0.18 ±0.74	0.13 ± 0.48	0 (0)	0 (0)	0.87
Occupational therapist	0.43 ± 1.99	0.71 ± 2.80	0 (0)	0(0)	0.01
Administrative	0.34 ± 1.17	0.34 ± 1.12	0 (0)	0 (0)	0.98
Unspecified	0.39 ± 1.43	0.22 ± 0.80	0 (0)	0 (0)	<0.01
Health Care Assistant	0.08 ± 0.55	0.08 ± 0.58	0 (0)	0 (0)	0.33
Clinical psychologist	0.19 ± 0.92	0.24 ± 1.06	0 (0)	0 (0)	0.03
Other therapist	0.02 ± 0.21	0.01 ± 0.18	0 (0)	0 (0)	0.21
Inpatient bed days	31.58 ± 51.99	21.09 ± 52.93	0 (47)	0 (11.5)	<0.001
Days active†	322.81 ± 95.63	363.48 ± 12.53	365 (0)	365 (0)	<0.001

* Testing the individual differences between the two periods using Wilcoxon signed-rank test
† Days active are calculated from the total number of days on the SLAM caseload during the relevant period – i.e. excluding days between SLAM referrals.

For peer review only

Table 4. Frequencies of service staff contacts during the 12 month periods before and after the index date – 'mirror' analysis excluding the influence of the time between paliperidone palmitate initiation and in-patient discharge (mirror approach 2).

Type of contact	Mean ± SD		Median (Interquartile range)		p-value*
	Before paliperidone palmitate initiation	After paliperidone palmitate initiation	Before paliperidone palmitate initiation	After paliperidone palmitate initiation	
Face-to-face contact					
Total	29.68 ± 24.85	25.79 ± 20.37	26 (24)	22 (17)	<0.001
Nurse	17.64 ± 16.89	15.25 ± 12.74	15 (22)	13(11)	<0.001
Social worker	3.96 ± 5.89	3.33 ± 6.12	2 (5)	1 (3)	<0.001
Medical staff	1.74 ± 2.97	1.16 ± 2.13	1 (2)	0 (1)	<0.001
Consultant	1.76 ± 2.57	1.53 ± 2.20	1 (2)	1 (2)	0.07
Occupational therapist	0.70 ± 2.51	0.94 ± 3.30	0 (0)	0 (0)	0.41
Administration	1.07 ± 2.90	0.97 ± 3.10	0 (1)	0 (1)	0.09
Unspecified	1.33 ± 4.66	0.91 ± 3.11	0 (1)	0 (0)	<0.001
HCA	0.83 ± 6.23	0.76 ± 3.69	0 (0)	0 (0)	0.34
Clinical psychologist	0.58 ± 2.61	0.85 ± 3.63	0 (0)	0 (0)	0.04
Other therapist	0.08 ± 0.77	0.08 ± 0.96	0 (0)	0 (0)	0.44

For peer review only - http://bmjopen.bmj.com/site/about/guidelines.xhtml

Phone contact					
Total	11.03 ± 10.45	8.74 ± 8.74	8 (13.5)	6 (11)	<0.001
Nurse	6.06 ± 7.41	4.89 ± 5.86	3 (8)	3 (6)	<0.001
Social worker	2.98 ± 5.73	2.01 ± 4.65	1 (3)	0 (2)	<0.001
Medical staff	0.21 ± 0.82	0.10 ± 0.40	0 (0)	0 (0)	<0.001
Consultant	0.18 ±0.74	0.13 ± 0.48	0 (0)	0 (0)	0.87
Occupational therapist	0.52 ± 2.22	0.71 ± 2.80	0 (0)	0 (0)	0.19
Administration	0.39 ± 1.20	0.34 ± 1.12	0 (0)	0 (0)	0.20
Unspecified	0.39 ± 1.43	0.22 ± 0.80	0 (0)	0 (0)	0.001
HCA	0.10 ± 0.60	0.08 ± 0.58	0 (0)	0 (0)	0.13
Clinical psychologist	0.19 ± 0.92	0.24 ± 1.06	0 (0)	0 (0)	0.03
Other therapist	0.02 ± 0.21	0.01 ± 0.18	0 (0)	0 (0)	0.21
Inpatient bed days	45.06±64.00	21.09±52.93	20 (61)	0 (11.5)	<0.001
Days active §	322.81±95.63	363.48±12.53	365 (0)	365 (0)	<0.001

* Testing individual differences between the two periods using Wilcoxon signed-rank test
§ Days active are calculated from the total number of days on the SLAM caseload during the relevant period- i.e. excluding days between SLAM referral.

For peer review only

For peer review only - http://bmjopen.bmj.com/site/about/guidelines.xhtml

Figure 1. a) The unadjusted model took into account all pre and post initiation time for both inpatient and outpatients. b) Mirror 1 approach- for inpatients all time between initiation and inpatient discharge was discarded, as well as an equivalent amount of time pre-initiation. c) Mirror 2 approach- for inpatients the time between initiation and inpatient discharge was discarded. Shaded area reflects inpatient time that was discarded.

For peer review only

For peer review only - http://bmjopen.bmj.com/site/about/guidelines.xhtml

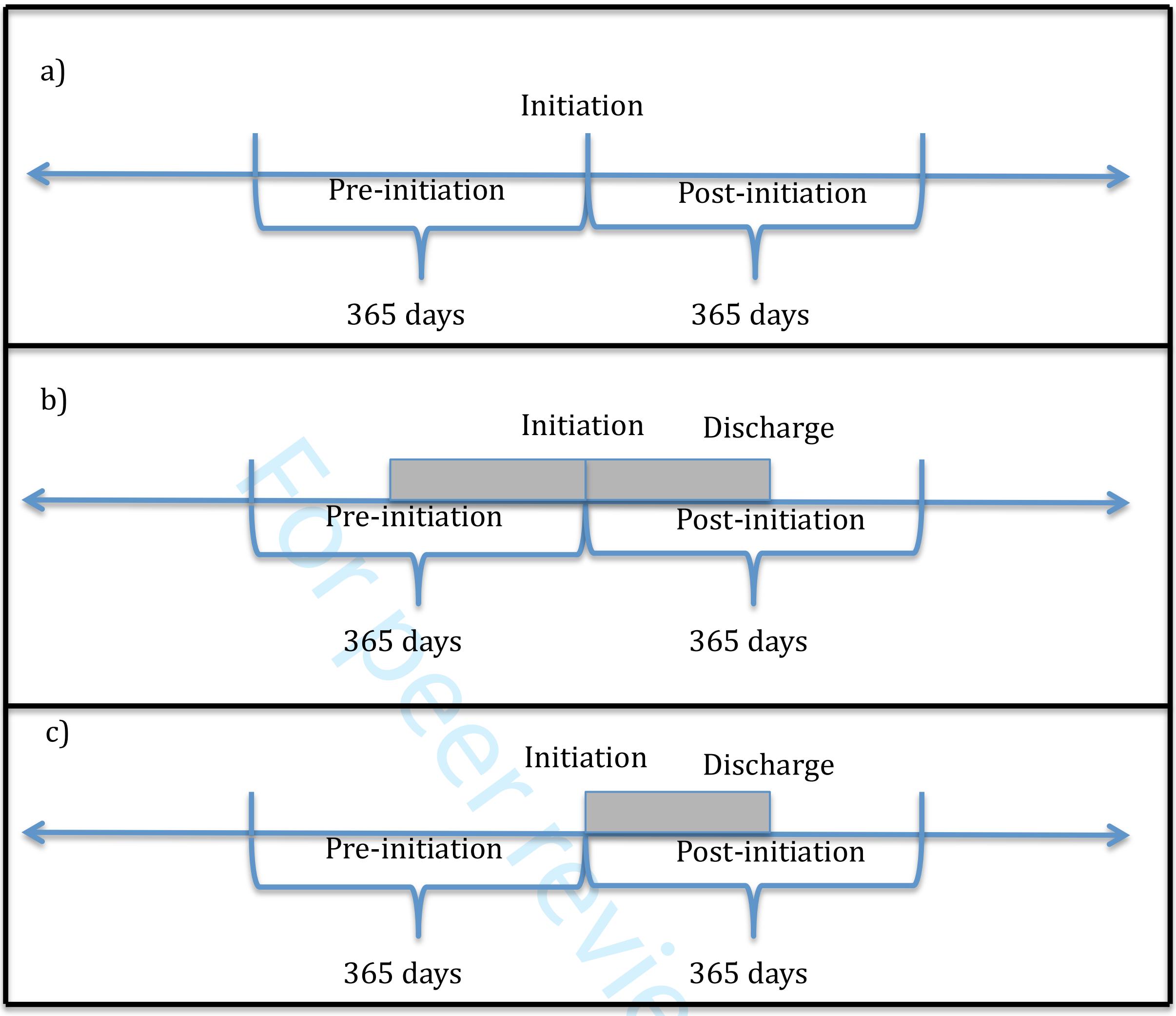

For peer review only

BMJ Open

Mental health care utilisation by patients before and after receiving paliperidone palmitate treatment: mirror-image analyses.

Journal:	*BMJ Open*
Manuscript ID	bmjopen-2021-051567.R1
Article Type:	Original research
Date Submitted by the Author:	14-Jan-2022
Complete List of Authors:	Kadra-Scalzo, Giouliana; King's College London<br>Ahn, Deborah; King's College London<br>Bird, Alex; Janssen Pharmaceutical Companies of Johnson and Johnson<br>Broadbent, Matthew; South London and Maudsley Mental Health NHS Trust<br>Chang, Chin-Kuo; King's College London; National Taiwan University, Institute of Epidemiology and Preventive Medicine, College of Public Health<br>Pritchard, Megan; South London and Maudsley Mental Health NHS Trust<br>Shetty, Hitesh; South London and Maudsley Mental Health NHS Trust<br>Taylor, David; South London and Maudsley NHS Foundation Trust<br>Hayes, Richard; King's College London<br>Stewart, Robert; King's College London; South London and Maudsley Mental Health NHS Trust
**Primary Subject Heading**:	Pharmacology and therapeutics
Secondary Subject Heading:	Evidence based practice, Mental health
Keywords:	MENTAL HEALTH, Adult psychiatry < PSYCHIATRY, Schizophrenia & psychotic disorders < PSYCHIATRY

SCHOLARONE™
Manuscripts

I, the Submitting Author has the right to grant and does grant on behalf of all authors of the Work (as defined in the below author licence), an exclusive licence and/or a non-exclusive licence for contributions from authors who are: i) UK Crown employees; ii) where BMJ has agreed a CC-BY licence shall apply, and/or iii) in accordance with the terms applicable for US Federal Government officers or employees acting as part of their official duties; on a worldwide, perpetual, irrevocable, royalty-free basis to BMJ Publishing Group Ltd ("BMJ") its licensees and where the relevant Journal is co-owned by BMJ to the co-owners of the Journal, to publish the Work in this journal and any other BMJ products and to exploit all rights, as set out in our licence.

The Submitting Author accepts and understands that any supply made under these terms is made by BMJ to the Submitting Author unless you are acting as an employee on behalf of your employer or a postgraduate student of an affiliated institution which is paying any applicable article publishing charge ("APC") for Open Access articles. Where the Submitting Author wishes to make the Work available on an Open Access basis (and intends to pay the relevant APC), the terms of reuse of such Open Access shall be governed by a Creative Commons licence – details of these licences and which Creative Commons licence will apply to this Work are set out in our licence referred to above.

Other than as permitted in any relevant BMJ Author's Self Archiving Policies, I confirm this Work has not been accepted for publication elsewhere, is not being considered for publication elsewhere and does not duplicate material already published. I confirm all authors consent to publication of this Work and authorise the granting of this licence.

For peer review only

Mental health care utilisation by patients before and after receiving paliperidone palmitate treatment: mirror-image analyses.

Giouliana Kadra-Scalzo[1], Deborah Ahn[1], Alex Bird[2], Matthew Broadbent[3], Chin-Kuo Chang[1,4,5], Megan Pritchard[1,3], Hitesh Shetty[3], David Taylor[3], Richard Hayes*[1] and Robert Stewart*[1,3]

[1] Institute of Psychiatry, Psychology and Neuroscience, King's College London, London, UK

[2] Janssen, UK

[3] South London and Maudsley NHS Foundation Trust, London, UK

[4] Institute of Epidemiology and Preventive Medicine, College of Public Health, National Taiwan University, Taipei City, Taiwan

[5] Global Health Program, College of Public Health, National Taiwan University, Taipei City, Taiwan

*Equal contributions to manuscript

Corresponding author: Dr. Giouliana Kadra-Scalzo, BRC Nucleus, Mapother House, De Crespigny Park, IOPPN, King's College London, London, SE5 8AF, UK; email: giouliana.kadra@kcl.ac.uk;

For peer review only

Abstract

Objectives: To compare mental healthcare use and health care professional (HCP) contacts for patients before and after initiation of paliperidone palmitate.

Setting: The South London and Maudsley NHS Foundation Trust (SLAM) Biomedical Research Centre Clinical Record Interactive Search (CRIS).

Participants: We identified all adults with a diagnosis of schizophrenia (ICD 10: F20.x), who had received paliperidone palmitate prescription for at least 365 days and had at least 1 year of recorded treatment from SLAM, prior to the first recorded receipt of paliperidone palmitate.

Primary and secondary outcome measures: Inpatient and community mental healthcare service use, such as inpatient bed days, number of active days in the service, face-to-face and telephone HCP use in the 12 months before and after paliperidone palmitate initiation.

Results: We identified 664 patients initiated on paliperidone palmitate. Following initiation, inpatient bed days were lower, although patients remained active on the service caseload for longer for both mirror approach 1 [mean difference of inpatient bed days -10.48 (95% CI: (-15.75)- (-5.22)); days active 40.67 (95% CI: 33.39-47.95)] and mirror approach 2 [mean difference of inpatient bed days -23.96 (95% CI: (-30.01)- (-17.92)); mean difference of days active is 40.69 (95% CI: 33.39-47.94)]. The post-initiation period was further characterised by fewer face-to-face and telephone contacts with medical and social work HCPs, and an increased contact with clinical psychologists.

For peer review only - http://bmjopen.bmj.com/site/about/guidelines.xhtml

Conclusions: Our findings indicate a change in the profile of HCP use, consistent with a transition from treatment to possible rehabilitation.

Strengths and Limitations of this study:

- We captured a large and diverse sample of patients in naturalistic settings, reflective of real-life clinical practice.
- We employed two methods of mirror image analyses, to address the potentially biasing impact of the inpatient episodes within which many initiation events take place.
- The study was observational in nature and therefore the before-after comparisons cannot be assumed to represent causal relationships.
- We were unable to examine the effects of factors, which were not captured by the routine health records.

For peer review only

For peer review only - http://bmjopen.bmj.com/site/about/guidelines.xhtml

Background

Treatment discontinuation and covert non-adherence is high amongst patients with serious mental illnesses (1) and current guidelines for schizophrenia recommend that depot medication can be considered (2) to reduce relapse. For patients who have been previously non-adherent, depot antipsychotics have been associated with lower risk of re-hospitalisation (3,4) and relapse (5) in comparison with oral preparations of the same medications and placebos (6). However, there have been significant concerns over extrapyramidal effects (7–9); and the introduction of second-generation (atypical) depot antipsychotics was cited as a potentially significant opportunity for a lower risk of movement disorders (10).

Research into second-generation depot antipsychotics has been relatively sparse. Findings from studies examining risperidone long-acting injection have been mixed with some evidence indicating that risperidone depot is associated with reduced hospital admissions, total in-patient days (11) and improvement in clinical symptoms (12), whereas other studies have suggested that risperidone depot either made no difference (13) or was associated with increased bed stay and healthcare costs (14).

Paliperidone palmitate was introduced in the UK National Health Service in 2011 and is a second-generation antipsychotic depot typically administered once a month intramuscularly. Several studies have investigated the clinical efficacy of paliperidone palmitate in treating schizophrenia symptoms (15,16). Although paliperidone palmitate is associated with higher acquisition cost in comparison to other oral antipsychotics (17), several observational studies have investigated its effectiveness and reported favourable findings in relation to in-patient hospitalization. For example, Bressington

For peer review only - http://bmjopen.bmj.com/site/about/guidelines.xhtml

and colleagues (18) reported a reduced number of acute in-patient admissions a year after paliperidone palmitate initiation. Similarly, Taylor and Olofinjana (19) reported a significant decrease in the number and length of hospital admissions per patient per year. Therefore, it appears that although paliperidone palmitate may be associated with a higher initial cost, this may be offset over time through a decrease in rehospitalisation. However, although hospitalisation episodes are strong determinants of healthcare costs (14), it is also important to understand health care professional (HCP) contacts, which reflect intensity of input required in outpatient settings. To the authors' knowledge, there has been no research to date that has examined secondary mental health care use in this level of detail. Consequently, the aim of this study was to describe inpatient and community mental healthcare service use, using observational data including HCP contacts, of patients with schizophrenia being treated with paliperidone palmitate, specifically comparing the frequency and duration of HCP contacts before and after initiation of paliperidone palmitate therapy, using mirror-image methodologies. On one hand, observational data are limited with respect to trial data because of the lack of randomisation and thus the risk of residual confounding; on the other hand, they provide an opportunity to assess associations in a more naturalistic and generalisable setting than is generally the case for trials.

For peer review only - http://bmjopen.bmj.com/site/about/guidelines.xhtml

Methods

The data for this study were sourced from the South London and Maudsley NHS Foundation Trust (SLAM) Biomedical Research Centre Clinical Record Interactive Search (CRIS). CRIS has been previously described in detail (20,21); briefly this is a data resource sourced from the electronic mental health records of SLAM, which provides comprehensive mental health services to a geographic catchment of 1.36 million residents in four boroughs of south London. All records are de-identified by CRIS and made available for research use under the governance framework (22). CRIS data have been substantially enhanced through the application of natural language processing to text fields (21).

We identified all adults with a diagnosis of schizophrenia (ICD 10: F20.x), who had received paliperidone palmitate prescription for at least 365 days and had at least 1 year of recorded treatment from SLAM, continuous or not, prior to the first recorded receipt of paliperidone palmitate. The first recorded receipt of paliperidone palmitate was defined as the 'index date' for definitions of outcomes and covariates and all patients were followed-up for one year from their index date. The observation period was from 2011 (when paliperidone palmitate was introduced in the UK) to 2016 (when data were extracted for this analysis).

A range of covariates were identified for cohort characterisation. These included age on the index date, gender, and recorded ethnicity grouped into the following categories: white, black, Asian or mixed/other. Marital status was derived from a clinician-completed compulsory structured field in the source record, and was grouped into the following categories: single; married / civil partnership / cohabiting; divorced / separated; widowed. Employment status was derived from compulsory

For peer review only - http://bmjopen.bmj.com/site/about/guidelines.xhtml

structured fields and referred to the most recent recording on or prior to the index date. Smoking status was derived from a combination of structured fields in the record and a natural language processing algorithm used to ascertain free-text inferring to smoking status (17); current or previous smoking in the record was ascertained prior to the index date. The Health of the Nation Outcome Scales (HoNOS) instrument is routinely used in SLAM services for monitoring purposes, and individual scales from this instrument were extracted as 5-point scores (no problem, minimal, mild, moderate and severe problem). HCP contacts were quantified from structured meta-data for SLAM case note entries, ascertaining contacts on the basis of an 'attended' encounter recorded as either face-to-face or by phone. The profession of the person posting each entry was ascertained from a SLAM Human Resources database of named staff mapped to professional groups. Within the intervals of interest, durations of time were also calculated that each individual spent as an inpatient, and 'active' to SLAM (i.e. receiving assessment/care and not discharged). The category of 'medical' refers to trainee psychiatrists.

Ethics approval

CRIS is approved for secondary analysis by the Oxfordshire Research Ethics Committee C (reference 18/SC/0372).

Patient and public involvement

The project was reviewed and approved by the CRIS Oversight Committee. All CRIS related research projects are considered and approved by a patient-led Oversight Committee, reporting to the Caldicott Guardian. The committee considers the appropriateness of the research proposals and adjudicates on risks of de-

For peer review only - http://bmjopen.bmj.com/site/about/guidelines.xhtml

anonymisation at the analysis planning stage.

Statistical analyses

We used STATA 13 for all statistical analyses. Sample characteristics were summarised for the total cohort. We compared HCP use between the 12 months before and after initiation, choosing this time period *a priori* due to the risk of selection (attrition) bias for longer post-initiation periods. Mean numbers of HCP contacts were compared between the two periods of interest within individuals in the sample using Wilcoxon signed-rank tests. The level of significance was a p value less than 0.05. The median and interquartile range (IQR) are also provided for all HCP contacts. Figure 1 describes all methodologies adopted to investigate the data: a) the unadjusted analysis reflects the unmodified pre-/post-initiation comparisons of HCP contacts; b) in mirror approach 1, for inpatients all time between initiation and inpatient discharge was discarded, as well as an equivalent amount of time pre-initiation to minimise potential bias from extra inpatient days required for paliperidone palmitate stabilisation, whilst ensuring that comparisons were made between periods of identical duration. For all outpatients no time was excluded; c) in mirror approach 2, for service users who were inpatients at the time of their paliperidone palmitate initiation, the time between paliperidone palmitate initiation and in-patient discharge was excluded, as this is the period when the new medication is stabilising. In the latter mirror image approach, the days between hospitalisation and paliperidone palmitate initiation are attributed to the 'before' period, following the assumption that the hospitalisation period is due to the treatment failure of the previous antipsychotic (19). For all outpatients no time was excluded.

For peer review only - http://bmjopen.bmj.com/site/about/guidelines.xhtml

Results

We identified 664 patients with schizophrenia who met the inclusion criteria; 375 (56.5%) had paliperidone palmitate initiated in inpatient settings and 289 (43.5%) had paliperidone palmitate initiated in outpatient settings. Of people with schizophrenia receiving paliperidone palmitate for at least a year, 39.5 % (n=262) were documented as receiving it for at least 3 years. Supplementary Table 1 describes the socio-demographic, socioeconomic and clinical symptoms characteristics of the cohort. The mean age was 43 years, 60% of the sample were male, 60% of black ethnicity, close to 80% were single, and over 80% were unemployed. Close to three quarters were recorded as being current smokers. From most recent HoNOS scores, significant overactive/agitated behaviour was present in around 30% of the sample at initiation, around 25% had problematic alcohol or substance use, around 20% had significant cognitive problems, over 60% had significant hallucinations and/or delusions; however, the prevalence of depressive and self-harm problems were both below 20%. Significant difficulties in social relationships were present in approximately 40%, impaired activities of daily living in around 30%, significant problems with living conditions in over 20%, and impaired occupational/recreational activity in around 35%.

Table 1 summarises the unmodified analyses comparing mental healthcare and HCP use before and after paliperidone palmitate initiation. Overall, face-to-face contacts were significantly lower after initiation for nurses [mean difference -2.38 (95%CI: (-3.69)- (-1.07)], social workers [mean difference -0.64 (95%CI: (-1.08)- (-0.19))], medical professionals [mean difference -0.58 (95%CI:(-0.81)- (-0.34))], and unspecified staff members [mean difference -0.42 (95%CI: (-0.76)- (-0.07))].

For peer review only - http://bmjopen.bmj.com/site/about/guidelines.xhtml

However, contact with clinical psychologists was increased in the post-initiation period [mean difference 0.27 (95%CI: (-0.01)-0.56)]. Contact with occupational therapists in the post-initiation period was also higher but difference did not reach statistical significance. Telephone HCP contacts showed similar differences. Inpatient bed days [mean difference 13.93 (95%CI: 7.31- 20.54)] and days 'active' to SLAM [mean difference 40.67 (95%CI: 33.39- 47.95)] were significantly higher during the post-initiation period.

Table 2 describes results for the first mirror image analysis, where paliperidone palmitate post-initiation inpatient period was excluded as well as an equivalent period pre-initiation. The post-initiation period was characterised with significantly lower face-to-face contact with social workers [mean difference -0.06 (95%CI: (-0.49)- 0.37)], medical professionals [mean difference -0.31 (95% CI: (-0.54)- (-0.08))] and unspecified professionals [mean difference -0.41 (95% CI: (-0.76)- (-0.07))]. The post-initiation period was also characterised with significantly lower telephone contact with social workers [mean difference -0.42 (95% CI: (-0.76)- (-0.07))], medical professionals [mean difference -0.08 (95% CI: (-0.13)- (-0.03))] and unspecified professionals [mean difference -0.17 (95% CI: (-0.29)- (-0.06))]. However, face-to-face and telephone contact with clinical psychologists [face-to-face mean difference 0.27 (95% CI: (-0.01)- 0.56); telephone mean difference 0.05 (95% CI: (-0.03)- 0.13)] was significantly higher than during the pre-initiation period. In the post-initiation period, telephone contact with occupational therapist was also significantly higher [mean difference 0.27 (95% CI: 0.05- 0.50)]. The post-initiation period was also characterised in this model by fewer inpatient bed days [mean

For peer review only - http://bmjopen.bmj.com/site/about/guidelines.xhtml

difference -10.48 (95% CI: (-15.74)- (-5.22))] and an increase in days active to SLAM services [mean difference 40.67 (95% CI: 33.39-47.94)].

Table 3 illustrates the analysis for the second mirror image approach, where the period between paliperidone palmitate initiation and in-patient discharge was excluded. Overall face-to-face contacts were lower after paliperidone palmitate initiation – this was statistically significant for nurses [mean difference -2.38 (95% CI: (-3.69)- (-1.07)], social workers [mean difference -0.64 (95% CI: (-1.08)- (-0.19))], medical professionals [mean difference -0.58 (95% CI: (-0.81)- (-0.34))], and unspecified staff members [mean difference -0.42 (95% CI: (-0.76)- (-0.07))]. In addition, telephone contacts were significantly lower after paliperidone palmitate initiation for nurses [mean difference -1.17 (95% CI: (-1.73)- (-0.60))], social workers [mean difference -0.97 (95% CI: (-1.35)- (-0.59))], medical staff [mean difference -0.11 (95% CI: (-0.17)- (-0.05))] and unspecified staff members [mean difference -0.17 (95% CI: (-0.29)- (-0.06))]. We further observed an increased face-to-face and telephone contact with clinical psychologists [mean difference -0.27 (95% CI: (-0.02)- 0.56), and mean difference 0.05 (95% CI: (-0.03)- 0.14), respectively]. There was also a significant decrease in inpatient bed days post-initiation [mean difference -23.96 (95% CI: (-30.01)- (-17.92))], and higher number of days 'active' to SLAM [mean difference 40.69 (95% CI: 33.39- 47.94)].

For peer review only - http://bmjopen.bmj.com/site/about/guidelines.xhtml

Discussion

To our knowledge, this is the first study to investigate HCP use in secondary mental healthcare associated with paliperidone palmitate initiation. In a large mental healthcare database, we found that the initiation of paliperidone palmitate was associated with significantly decreased face-to-face and telephone contact with medical professionals, social workers and unspecified HCP and increased contact with therapeutic services such as clinical psychologists. This, in addition to the higher proportion of time spent 'active' in SLAM after initiation, and a decrease in days spend as an inpatient is consistent with a shift from treatment and risk management to rehabilitation. The results were largely consistent irrespective of the mirror image approach that was employed to take into account the potentially biasing effects of inpatient care at the time of treatment initiation, although reduction in nursing input post-initiation was only evident in the unadjusted analysis and second mirror image approach (but not in mirror image approach one).

Considering hospitalisation, our results are consistent with previous research findings that paliperidone palmitate initiation is associated with a reduction in inpatient bed days after the two mirror approaches were applied (19,23); we were able to replicate this in a larger cohort of patients initiated on paliperidone palmitate than previously reported, as well as using within-individual comparisons that will have removed the influence of between-individual differences as confounding factors. Taylor and Olofinjana (19) have argued that there are several possible mechanisms that may explain this assumed effect of paliperidone palmitate, including the ability to give loading doses and higher equivalent doses compared to risperidone depot. However, research comparing paliperidone palmitate to other long acting injectables

For peer review only - http://bmjopen.bmj.com/site/about/guidelines.xhtml

such as aripiprazole and haloperidol, have reported that paliperidone palmitate is prescribed at a lower dose and after 12months has a similar reduction in urgent consultation, psychiatric hospitalisations and psychiatric symptoms (24). Ultimately a formal randomised controlled trial would be required to assess this.

The post-initiation reduction in contact with medical and social work staff in both models, and reduction in nursing staff contact in the second model, could reflect improvements in mental state and is consistent with a wider benefit of treatment initiation than is indicated by inpatient care comparisons. Furthermore, the increased contact with clinical psychology staff does not support an overall reduction in HCP contact but instead an altered profile of contact from staff members responsible for treatment compared to those responsible for rehabilitation. It should be borne in mind that no control condition was included in the design – i.e. the apparent HCP changes might have been observed regardless of the date chosen to divide comparison periods, and/or samples receiving inpatient care may naturally have higher medical and social work involvement prior to an admission episode. Furthermore, if changes in HCP contacts represent an effect of the intervention rather than inpatient care, it was not possible to subdivide the sample in terms of conditions prior to the event (i.e. the precise medication change being instituted). Further research could benefit from examining this in the future. Findings from previous observational research have indicated that as compared to risperidone long-acting injectables, patients prescribed paliperidone palmitate are more likely to be adherent to their treatment, to have reduced hospitalisation and fewer emergency department visits (25). Furthermore, in France and Sweden, paliperidone palmitate has been found to be more cost-effective and to have a lower cost over 5 years, as

For peer review only - http://bmjopen.bmj.com/site/about/guidelines.xhtml

compared to other antipsychotics such as risperidone, aripiprazole and haloperidol (26,27).

This study had several strengths. We examined a large and diverse sample of patients in naturalistic settings. Therefore, our sample should be maximally reflective of health care professional contact in real-world clinical settings. The use of mental healthcare data was justified because very few paliperidone palmitate initiations would be expected outside specialist services. Furthermore, we had sufficient statistical power to characterise the sample according to a diverse number of patient characteristics, and the within-patient comparisons removes the effect of between-patient differences as confounding factors. Finally, in addition to the unadjusted analysis, we employed two methods of mirror image analyses, to address the potentially biasing impact of the inpatient episodes within which many initiation events took place.

On the other hand, several limitations need to be borne in mind. The study was observational in nature and therefore the before-after comparisons cannot be assumed to represent causal relationships (i.e. a treatment effect). In addition, the data were limited by availability from a routine health record and therefore we were unable to examine factors underlying changes in HCP contacts, such as clinical symptoms before and after paliperidone palmitate and prior medication history. In addition, selecting patients who have taken paliperidone palmitate for a year, are likely to represent a group of patients who have good outcomes. Mace and colleagues (28) have indicated that patients with longer duration of illness, who have been initiated on long-acting injectables such as haloperidol deconate, for reasons other than non-adherence, are more likely to remain on their treatment. Furthermore,

For peer review only

we did not examine the formulation of paliperidone palmitate (e.g. monthly; three-monthly), however at the time the study was conducted we believe the monthly formulation was mostly in use. In addition, a longer follow-up period would have allowed us to investigate longer-term effect of paliperidone palmitate and therefore should be considered by further research. It is also important to consider that follow-up began at the point paliperidone palmitate was initiated as opposed to hospital discharge. Furthermore, we did not include a control group of another depot or oral antipsychotic, therefore we cannot infer that changes associated with paliperidone palmitate initiation would not have been observed with other interventions. Lastly, we did not make adjustments for multiple comparisons in the analyses.

Overall, our findings suggest that paliperidone palmitate initiation is associated with a change in healthcare professional contact which could indicate change in patients' presentation. However, further research is needed that focuses on examining changes in mental health symptoms from the before to after period to support this hypothesis. Finally, we did not seek to quantify the economic impact of changes in health service use, and further research would be needed to determine whether this translates into lower overall service costs.

For peer review only
For peer review only - http://bmjopen.bmj.com/site/about/guidelines.xhtml

Authors' contribution:

GKS drafted the manuscript and together with DA conducted the analyses. MB, MP and HS put together the data extraction plan and extracted the data. AB, CK-C, DT, RH and RS contributed to the conceptualization and designing the study. All authors critically revised the manuscript and have approved the final version.

Competing interests: RDH, C-KC, HS and RS have received research funding from Roche, Pfizer, Janssen and Lundbeck. GKS has received funding from Janssen and Lundbeck. RS has also received research funding from Takeda. AB is an employee of Janssen, UK. DT has received investigator-initiated research grants from Astra-Zeneca, Eli Lilly, Janssen, Lundbeck, Otsuka, Servier, Sunovion.

Funding: The study described here was funded by Janssen UK. This work was supported by the Clinical Record Interactive Search (CRIS) platform funded and developed by the National Institute for Health Research (NIHR) Biomedical Research Centre at South London and Maudsley NHS Foundation Trust and King's College London, and a joint infrastructure grant from Guy's and St Thomas' Charity and the Maudsley Charity (grant number BRC-2011-10035). RDH, RS, HS, C-KC, RDH and GKS receive salary support from the National Institute for Health Research (NIHR) Biomedical Research Centre at South London and Maudsley NHS Foundation Trust and King's College London. DT is a pharmacist and clinical academic at the South London and Maudsley Hospital NHS Trust and King's College London. The views expressed are those of the authors and not necessarily those of the NHS, the NIHR or the Department of Health.

For peer review only - http://bmjopen.bmj.com/site/about/guidelines.xhtml

Data availability: The data that support the findings of this study are available on request from the corresponding author. The data are not publicly available due to the Information Governance framework and REC approval in place concerning CRIS data use.

For peer review only

For peer review only - http://bmjopen.bmj.com/site/about/guidelines.xhtml

References

1. Tiihonen J, Haukka J, Taylor M, Haddad PM, Patel MX, Korhonen P. A nationwide cohort study of oral and depot antipsychotics after first hospitalization for schizophrenia. American Journal of Psychiatry. 2011;168(6):603–9.

2. NICE, NCCMH. Schizophrenia. THE NICE GUIDELINE ON CORE INTERVENTIONS IN THE TREATMENT AND MANAGEMENT OF SCHIZOPHRENIA IN ADULTS IN PRIMARY AND SECONDARY CARE . NICE; 2013.

3. Taylor D, Olofinjana O. Long-acting paliperidone palmitate - Interim results of an observational study of its effect on hospitalization. International Clinical Psychopharmacology. 2014;29(4):229–34.

4. Tiihonen J, Haukka J, Taylor M, Haddad PM, Patel MX, Korhonen P. A nationwide cohort study of oral and depot antipsychotics after first hospitalization for schizophrenia. American Journal of Psychiatry. 2011;168(6):603–9.

5. Leucht C, Heres S, Kane JM, Kissling W, Davis JM, Leucht S. Oral versus depot antipsychotic drugs for schizophrenia-A critical systematic review and meta-analysis of randomised long-term trials. Schizophrenia Research. 2011;127(1–3):83–92.

6. Kozma CM, Slaton T, Dirani R, Fastenau J, Gopal S, Hough D. Changes in schizophrenia-related hospitalization and ER use among patients receiving paliperidone palmitate: results from a clinical trial with a 52-week open-label extension (OLE). Current Medical Research and Opinion. 2011;27(8):1603–11.

7. Leucht S, Pitschel-Walz G, Abraham D, Kissling W. Efficacy and extrapyramidal side-effects of the new antipsychotics olanzapine, quetiapine, risperidone, and sertindole compared to conventional antipsychotics and placebo. A meta-analysis of randomized controlled trials. Schizophrenia Research. 1999;35(1):51–68.

8. Barnes TRE, Cursori DA. Long Term Depot Antipsychotics: A Risk-Benefit Assessment. Drug Safety. 1994;10(6):464–79.

9. Novick D, Haro JM, Perrin E, Suarez D, Texeira JM. Tolerability of outpatient antipsychotic treatment: 36-month results from the European Schizophrenia Outpatient Health Outcomes (SOHO) study. European Neuropsychopharmacology. 2009;19(8):542–50.

10. Leucht S, Pitschel-Walz G, Abraham D, Kissling W. Efficacy and extrapyramidal side-effects of the new antipsychotics olanzapine, quetiapine, risperidone, and sertindole compared to conventional antipsychotics and placebo. A meta-analysis of randomized controlled trials. Schizophrenia Research. 1999;35(1):51–68.

11. Niaz OS, Haddad PM. Thirty-five months experience of risperidonelong-acting injection in a UK psychiatric service including a mirror-image analysis of in-patient care. Acta Psychiatrica Scandinavica. 2007;116(1):36–46.

For peer review only - http://bmjopen.bmj.com/site/about/guidelines.xhtml

12. Taylor DM, Young C, Patel MX. Prospective 6-month follow-up of patients prescribed risperidone long-acting injection: Factors predicting favourable outcome. International Journal of Neuropsychopharmacology. 2006;9(6):685–94.

13. Taylor D, Fischetti C, Sparshatt A, Thomas A, Bishara D, Cornelius V. Risperidone long-acting injection: A 6-year mirror-image study of healthcare resource use. Acta Psychiatrica Scandinavica. 2009;120(2):97–101.

14. Young CL, Taylor DM. Health resource utilization associated with switching to risperidone long-acting injection. Acta Psychiatrica Scandinavica. 2006;114(1):14–20.

15. Hough D, Gopal S, Vijapurkar U, Lim P, Morozova M, Eerdekens M. Paliperidone palmitate maintenance treatment in delaying the time-to-relapse in patients with schizophrenia: A randomized, double-blind, placebo-controlled study. Schizophrenia Research. 2010;116(2–3):107–17.

16. Gopal S, Vijapurkar U, Lim P, Morozova M, Eerdekens M, Hough D. A 52-week open-label study of the safety and tolerability of paliperidone palmitate in patients with schizophrenia. Journal of Psychopharmacology. 2011;25(5):685–97.

17. Lefebvre P, Muser E, Duh MS, DerSarkissian M, Faust E, Kageleiry A, et al. Impact of paliperidone palmitate versus oral atypical antipsychotics on healthcare resource use and costs in veterans with schizophrenia. Amcp 2015. 2015;4:579–92.

18. Bressington D, Stock J, Hulbert S, MacInnes D. A retrospective observational study of the effectiveness of paliperidone palmitate on acute inpatient hospitalization rates. International Clinical Psychopharmacology. 2015;30(4):230–6.

19. Taylor D, Olofinjana O. Long-acting paliperidone palmitate - Interim results of an observational study of its effect on hospitalization. International Clinical Psychopharmacology. 2014;29(4):229–34.

20. Perera G, Broadbent M, Callard F, Chang C-K, Downs J, Dutta R, et al. Cohort profile of the South London and Maudsley NHS Foundation Trust Biomedical Research Centre (SLaM BRC) Case Register: Current status and recent enhancement of an Electronic Mental Health Record-derived data resource. BMJ Open. 2016;6(3).

21. Stewart R, Soremekun M, Perera G, Broadbent M, Callard F, Denis M, et al. The South London and Maudsley NHS Foundation Trust Biomedical Research Centre (SLAM BRC) case register: development and descriptive data. BMC Psychiatry. 2009;9(1):51.

22. Fernandes AC, Cloete D, Broadbent MTM, Hayes RD, Chang C-K, Jackson RG, et al. Development and evaluation of a de-identification procedure for a case register sourced from mental health electronic records. BMC medical informatics and decision making. 2013;13(1):71.

For peer review only

For peer review only - http://bmjopen.bmj.com/site/about/guidelines.xhtml

23. Bressington D, Stock J, Hulbert S, MacInnes D. A retrospective observational study of the effectiveness of paliperidone palmitate on acute inpatient hospitalization rates. International Clinical Psychopharmacology. 2015;30(4):230–6.

24. di Lorenzo R, Ferri P, Cameli M, Rovesti S, Piemonte C. Effectiveness of 1-year treatment with long-acting formulation of aripiprazole, haloperidol, or paliperidone in patients with schizophrenia: Retrospective study in a real-world clinical setting. Neuropsychiatric Disease and Treatment. 2019;15:183–98.

25. Joshi K, Pan X, Wang R, Yang E, Benson C. Healthcare resource utilization of second-generation long-acting injectable antipsychotics in schizophrenia: risperidone versus paliperidone palmitate. Current Medical Research and Opinion. 2016 Nov 1;32(11):1873–81.

26. Druais S, Doutriaux A, Cognet M, Godet A, Lançon C, Levy P, et al. Cost Effectiveness of Paliperidone Long-Acting Injectable Versus Other Antipsychotics for the Maintenance Treatment of Schizophrenia in France. PharmacoEconomics. 2016 Apr 1;34(4):363–91.

27. Mehnert A, Nicholl D, Pudas H, Martin M, McGuire A. Cost effectiveness of paliperidone palmitate versus risperidone long-Acting injectable and olanzapine pamoate for the treatment of patients with schizophrenia in Sweden. Journal of Medical Economics. 2012 Oct;15(5):844–61.

28. Mace S, Dzahini O, O'Hagan M, Taylor D. Haloperidol decanoate long-acting injection (HDLAI): Results of a 1-year mirror-image study. Therapeutic Advances in Psychopharmacology. 2018;8(9):241–9.

For peer review only

For peer review only - http://bmjopen.bmj.com/site/about/guidelines.xhtml

Table 1. Frequencies of health care professionals contacts during the 12 month periods before and after the index date – unmodified analysis of the total time periods

Type of contact	Mean ± SD number of contacts		Median (Interquartile range)		P-value*
	Before paliperidone palmitate initiation	After paliperidone palmitate initiation	Before paliperidone palmitate initiation	After paliperidone palmitate initiation	
Face-to-face contact					
Total	29.70 ± 24.90	25.8 ± 20.40	26 (24)	22 (17)	<0.001
Nurse	17.60 ± 16.90	15.3 ± 12.74	15 (22)	13 (11)	<0.001
Social worker	4.00 ± 5.90	3.32 ± 6.12	2 (5)	1 (3)	<0.001
Medical	1.74 ± 3.00	1.16 ± 2.13	1 (2)	0 (1)	<0.001
Consultant	1.76 ± 2.57	1.53 ± 2.20	1 (2)	1 (2)	0.07
Occupational therapist	0.70 ± 2.51	0.94 ± 3.30	0 (0)	0 (0)	0.41
Administrative	1.07 ± 2.90	0.97 ± 3.10	0 (1)	0 (1)	0.09
Unspecified	1.33 ± 4.66	0.91 ± 3.11	0 (1)	0 (0)	<0.001
Health Care Assistant	0.83 ± 6.23	0.76 ± 3.69	0 (0)	0 (0)	0.34
Clinical psychologist	0.58 ± 2.61	0.85 ± 3.63	0 (0)	0 (0)	0.04

For peer review only - http://bmjopen.bmj.com/site/about/guidelines.xhtml

Other therapist	0.08 ± 0.77	0.08 ± 0.96	0 (0)	0 (0)	0.44
Phone contact					
Total	11.03 ± 10.45	8.74 ± 8.74	8 (13.5)	6 (11)	<0.001
Nurse	6.06 ± 7.41	4.89 ± 5.86	3 (8)	3 (6)	<0.001
Social worker	2.98 ± 5.73	2.01 ± 4.65	1 (3)	0 (2)	<0.001
Medic	0.21 ± 0.82	0.10 ± 0.40	0 (0)	0 (0)	0.001
Consultant	0.18 ±0.74	0.13 ± 0.48	0 (0)	0 (0)	0.87
Occupational therapist	0.52 ± 2.22	0.71 ± 2.80	0 (0)	0 (0)	0.19
Administrative	0.39 ± 1.20	0.34 ± 1.12	0 (0)	0 (0)	0.20
Unspecified	0.39 ± 1.43	0.22 ± 0.80	0 (0)	0 (0)	0.001
Health Care Assistant	0.10 ± 0.60	0.08 ± 0.58	0 (0)	0 (0)	0.13
Clinical psychologist	0.19 ± 0.92	0.24 ± 1.06	0(0)	0 (0)	0.03
Other therapist	0.02 ± 0.21	0.01 ± 0.18	0 (0)	0(0)	0.21
Inpatient bed days	45.06 ± 64.00	58.99 ± 87.84	20 (61)	23.5 (79.5)	0.01
Days active†	322.81 ± 95.63	363.28 ± 13.54	365 (0)	365 (0)	<0.001

* Testing the individual differences between the two periods using Wilcoxon signed-rank test
† Days active are calculated from the total number of days on the SLAM caseload during the relevant period – i.e. excluding days between SLAM referrals

For peer review only

Table 2. Frequencies of health care professionals contacts during the 12 month periods before and after the index date – 'mirror' analysis excluding the influence of the inpatient period around paliperidone palmitate initiation (mirror approach 1)

Type of contact	Mean ± SD number of contacts		Median (Interquartile range)		P-value*
	Before paliperidone palmitate initiation	After paliperidone palmitate initiation	Before paliperidone palmitate initiation	After paliperidone palmitate initiation	
Face-to-face contact					
Total	26.87 ± 24.92	25.79 ± 20.37	23 (24)	22 (17)	0.29
Nurse	16.25 ± 16.62	15.25 ± 12.74	13 (21)	13 (11)	0.40
Social worker	3.39 ± 5.58	3.33 ± 6.12	1 (4)	1 (3)	0.03
Medical staff	1.47 ± 2.86	1.16 ± 2.13	0 (2)	0 (1)	<0.01
Consultant	1.76 ± 2.57	1.53 ± 2.20	1 (2)	1 (2)	0.07
Occupational therapist	0.62 ± 2.34	0.94 ± 3.30	0 (0)	0(0)	0.09
Administrative	0.97 ± 2.81	0.97 ± 3.09	0 (1)	0 (1)	0.59
Unspecified	1.33 ± 4.66	0.91 ± 3.11	0 (1)	0 (0)	<0.001
Health Care Assistant	0.77 ± 6.14	0.76 ± 3.69	0(0)	0(0)	0.07
Clinical psychologist	0.58 ± 2.61	0.85 ± 3.63	0(0)	0(0)	0.04

For peer review only - http://bmjopen.bmj.com/site/about/guidelines.xhtml

Other therapist	0.07 ± 0.77	0.08 ± 0.96	0(0)	0(0)	0.44
Phone contact					
Total	9.38 ± 9.57	8.74 ± 8.74	6.5 (12)	6 (11)	0.03
Nurse	5.28 ± 6.71	4.89 ± 5.86	3 (8)	3 (6)	0.29
Social worker	2.43 ± 5.18	2.01 ± 4.65	1 (2)	0 (2)	<0.001
Medical staff	0.18 ± 0.75	0.10 ± 0.40	0 (0)	0 (0)	0.02
Consultant	0.18 ±0.74	0.13 ± 0.48	0 (0)	0 (0)	0.87
Occupational therapist	0.43 ± 1.99	0.71 ± 2.80	0 (0)	0(0)	0.01
Administrative	0.34 ± 1.17	0.34 ± 1.12	0 (0)	0 (0)	0.98
Unspecified	0.39 ± 1.43	0.22 ± 0.80	0 (0)	0 (0)	<0.01
Health Care Assistant	0.08 ± 0.55	0.08 ± 0.58	0 (0)	0 (0)	0.33
Clinical psychologist	0.19 ± 0.92	0.24 ± 1.06	0 (0)	0 (0)	0.03
Other therapist	0.02 ± 0.21	0.01 ± 0.18	0 (0)	0 (0)	0.21
Inpatient bed days	31.58 ± 51.99	21.09 ± 52.93	0 (47)	0 (11.5)	<0.001
Days active†	322.81 ± 95.63	363.48 ± 12.53	365 (0)	365 (0)	<0.001

* Testing the individual differences between the two periods using Wilcoxon signed-rank test
† Days active are calculated from the total number of days on the SLAM caseload during the relevant period – i.e. excluding days between SLAM referrals.

For peer review only - http://bmjopen.bmj.com/site/about/guidelines.xhtml

Table 3. Frequencies of service staff contacts during the 12 month periods before and after the index date – 'mirror' analysis excluding the influence of the time between paliperidone palmitate initiation and in-patient discharge (mirror approach 2).

Type of contact	Mean ± SD		Median (Interquartile range)		p-value*
	Before paliperidone palmitate initiation	After paliperidone palmitate initiation	Before paliperidone palmitate initiation	After paliperidone palmitate initiation	
Face-to-face contact					
Total	29.68 ± 24.85	25.79 ± 20.37	26 (24)	22 (17)	<0.001
Nurse	17.64 ± 16.89	15.25 ± 12.74	15 (22)	13(11)	<0.001
Social worker	3.96 ± 5.89	3.33 ± 6.12	2 (5)	1 (3)	<0.001
Medical staff	1.74 ± 2.97	1.16 ± 2.13	1 (2)	0 (1)	<0.001
Consultant	1.76 ± 2.57	1.53 ± 2.20	1 (2)	1 (2)	0.07
Occupational therapist	0.70 ± 2.51	0.94 ± 3.30	0 (0)	0 (0)	0.41
Administration	1.07 ± 2.90	0.97 ± 3.10	0 (1)	0 (1)	0.09
Unspecified	1.33 ± 4.66	0.91 ± 3.11	0 (1)	0 (0)	<0.001
HCA	0.83 ± 6.23	0.76 ± 3.69	0 (0)	0 (0)	0.34
Clinical psychologist	0.58 ± 2.61	0.85 ± 3.63	0 (0)	0 (0)	0.04
Other therapist	0.08 ± 0.77	0.08 ± 0.96	0 (0)	0 (0)	0.44

For peer review only - http://bmjopen.bmj.com/site/about/guidelines.xhtml

Phone contact					
Total	11.03 ± 10.45	8.74 ± 8.74	8 (13.5)	6 (11)	<0.001
Nurse	6.06 ± 7.41	4.89 ± 5.86	3 (8)	3 (6)	<0.001
Social worker	2.98 ± 5.73	2.01 ± 4.65	1 (3)	0 (2)	<0.001
Medical staff	0.21 ± 0.82	0.10 ± 0.40	0 (0)	0 (0)	<0.001
Consultant	0.18 ±0.74	0.13 ± 0.48	0 (0)	0 (0)	0.87
Occupational therapist	0.52 ± 2.22	0.71 ± 2.80	0 (0)	0 (0)	0.19
Administration	0.39 ± 1.20	0.34 ± 1.12	0 (0)	0 (0)	0.20
Unspecified	0.39 ± 1.43	0.22 ± 0.80	0 (0)	0 (0)	0.001
HCA	0.10 ± 0.60	0.08 ± 0.58	0 (0)	0 (0)	0.13
Clinical psychologist	0.19 ± 0.92	0.24 ± 1.06	0 (0)	0 (0)	0.03
Other therapist	0.02 ± 0.21	0.01 ± 0.18	0 (0)	0 (0)	0.21
Inpatient bed days	45.06±64.00	21.09±52.93	20 (61)	0 (11.5)	<0.001
Days active §	322.81±95.63	363.48±12.53	365 (0)	365 (0)	<0.001

* Testing individual differences between the two periods using Wilcoxon signed-rank test
§ Days active are calculated from the total number of days on the SLAM caseload during the relevant period- i.e. excluding days between SLAM referral.

For peer review only - http://bmjopen.bmj.com/site/about/guidelines.xhtml

Figure 1. a) The unadjusted model took into account all pre and post initiation time for both inpatient and outpatients. b) Mirror 1 approach- for inpatients all time between initiation and inpatient discharge was discarded, as well as an equivalent amount of time pre-initiation. c) Mirror 2 approach- for inpatients the time between initiation and inpatient discharge was discarded. Shaded area reflects inpatient time that was discarded.

For peer review only

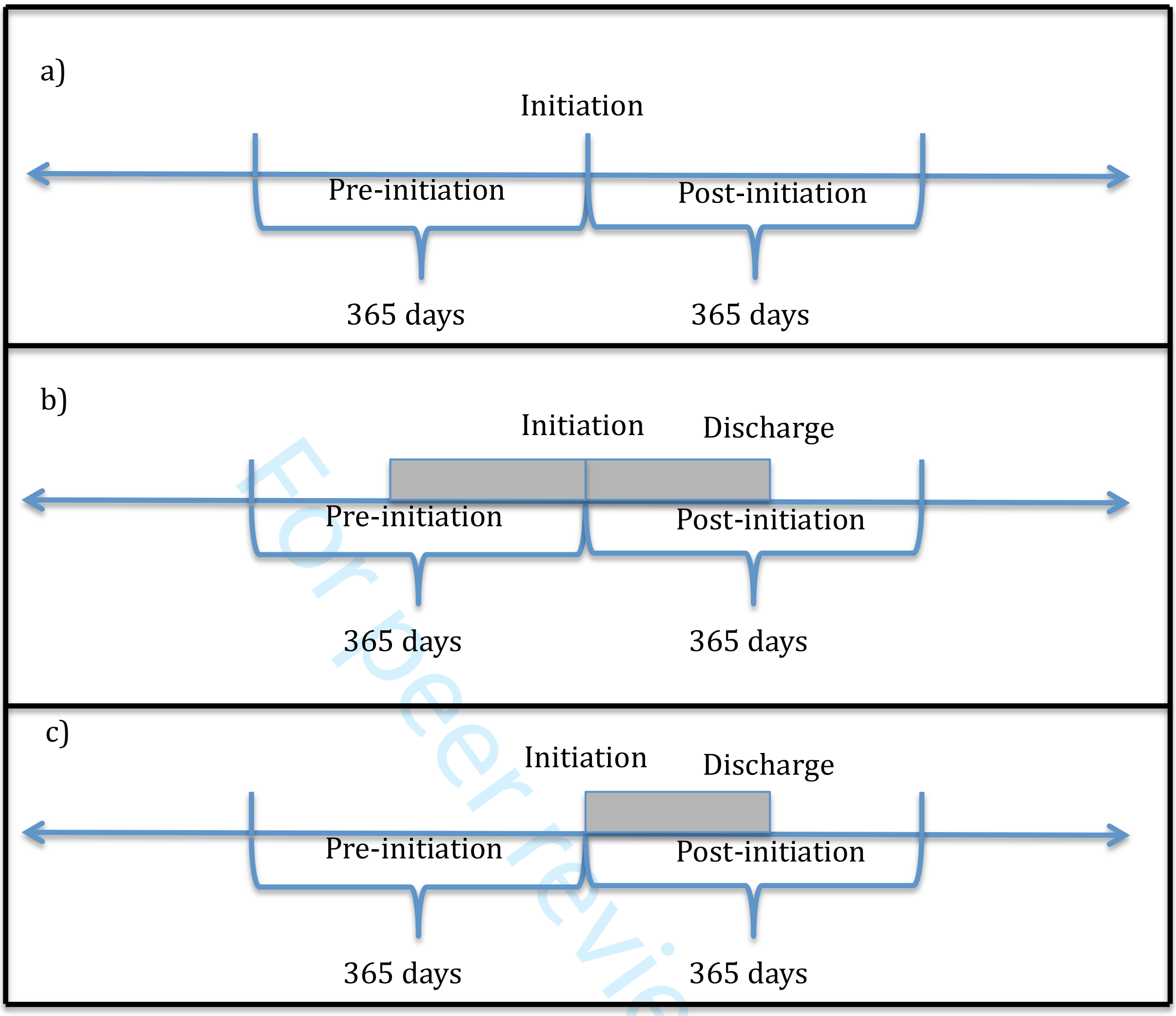

For peer review only

Supplementary Table 1. Sample characteristics (N=664)

Variables	Number (%) / Mean ± SD
Mean age at index date	42.7 ± 12.8
Gender	
Female	265 (39.9)
Male	399 (60.1)
Most recent employment status at index date	
Unemployed	549 (82.7)
Employed	22 (3.3)
Sick	8 (1.2)
Other	22 (3.3)
Missing	63 (9.5)
Most recent employment status 1 year after index date	
Unemployed	555 (83.6)
Employed	24 (3.6)
Sick	27 (4.1)
Other	36 (5.4)
Missing	22 (3.3)
Current or previous smoker at index date	
No	179 (27.0)
Yes	485 (73.0)
Ethnicity	
White	179 27.0)
Black	399 (60.1)
Asian	36 (5.4)
Other/mixed	50 (7.5)
Marital Status	
Single	521 (78.5)
Married/civil partner/cohabitating	54 (8.1)
Divorced/separated	80 (12.1)
Widowed	9 (1.4)

For peer review only

Most recent Health of the Nation Outcomes Scales (HoNOS) score prior to index date	
Overactive, agitated behaviour	
no problem	315 (48.2)
sub-threshold problem	147 (22.5)
mild but definitely present	103 (15.8)
moderately severe	53 (8.1)
severe to very severe	36 (5.5)
Non-accidental self-injury	
no problem	594 (90.8)
sub-threshold problem	39 (6.0)
mild but definitely present	12 (1.8)
moderately severe	4 (0.6)
severe to very severe	5 (0.8)
Problem-drinking or drug-taking	
no problem	397 (62.0)
sub-threshold problem	82 (12.8)
mild but definitely present	84 (13.1)
moderately severe	57 (8.9)
severe to very severe	20 (3.1)
Cognitive problems	
no problem	354 (54.1)
sub-threshold problem	165 (25.2)
mild but definitely present	90 (13.8)
moderately severe	38 (5.8)
severe to very severe	7 (1.1)
Physical illness or disability problems	
no problem	407 (62.4)
sub-threshold problem	129 (19.8)
mild but definitely present	83 (12.7)
moderately severe	30 (4.6)
severe to very severe	3 (0.5)
Hallucinations and delusions	

For peer review only

no problem	123 (18.9)
sub-threshold problem	128 (19.6)
mild but definitely present	186 (28.5)
moderately severe	145 (22.2)
severe to very severe	70 (10.7)
Depressed mood	
no problem	334 (51.3)
sub-threshold problem	200 (30.7)
mild but definitely present	94 (14.4)
moderately severe	16 (2.5)
severe to very severe	7 (1.1)
Other mental and behavioural problems	
no problem	176 (27.0)
sub-threshold problem	153 (23.5)
mild but definitely present	223 (34.2)
moderately severe	80 (12.3)
severe to very severe	20 (3.1)
Social relationships	
no problem	198 (30.5)
sub-threshold problem	195 (30.1)
mild but definitely present	174 (26.8)
moderately severe	66 (10.2)
severe to very severe	16 (2.5)
Activities of daily living	
no problem	263 (40.5)
sub-threshold problem	183 (28.2)
mild but definitely present	132 (20.3)
moderately severe	58 (8.9)
severe to very severe	14 (2.2)
Standard of living conditions	
no problem	353 (55.9)
sub-threshold problem	137 (21.7)
mild but definitely present	84 (13.3)

For peer review only

moderately severe	35 (5.5)
severe to very severe	23 (3.6)
Occupational and recreational activities	
no problem	235 (37.0)
sub-threshold problem	185 (29.1)
mild but definitely present	150 (23.6)
moderately severe	49 (7.7)
severe to very severe	17 (2.7)

For peer review only

STROBE Statement—checklist of items that should be included in reports of observational studies

	Item No.	Recommendation	Page No.
**Title and abstract**	1	(*a*) Indicate the study's design with a commonly used term in the title or the abstract	2
		(*b*) Provide in the abstract an informative and balanced summary of what was done and what was found	2
**Introduction**			
Background/rationale	2	Explain the scientific background and rationale for the investigation being reported	4
Objectives	3	State specific objectives, including any prespecified hypotheses	5
**Methods**			
Study design	4	Present key elements of study design early in the paper	6
Setting	5	Describe the setting, locations, and relevant dates, including periods of recruitment, exposure, follow-up, and data collection	6
Participants	6	(*a*) *Cohort study*—Give the eligibility criteria, and the sources and methods of selection of participants. Describe methods of follow-up<br>*Case-control study*—Give the eligibility criteria, and the sources and methods of case ascertainment and control selection. Give the rationale for the choice of cases and controls<br>*Cross-sectional study*—Give the eligibility criteria, and the sources and methods of selection of participants	6
		(*b*) *Cohort study*—For matched studies, give matching criteria and number of exposed and unexposed<br>*Case-control study*—For matched studies, give matching criteria and the number of controls per case	
Variables	7	Clearly define all outcomes, exposures, predictors, potential confounders, and effect modifiers. Give diagnostic criteria, if applicable	6,7
Data sources/ measurement	8*	For each variable of interest, give sources of data and details of methods of assessment (measurement). Describe comparability of assessment methods if there is more than one group	6,7
Bias	9	Describe any efforts to address potential sources of bias	8
Study size	10	Explain how the study size was arrived at	8

Continued on next page

For peer review only

Quantitative variables	11	Explain how quantitative variables were handled in the analyses. If applicable, describe which groupings were chosen and why	7, 8
Statistical methods	12	(*a*) Describe all statistical methods, including those used to control for confounding	8
		(*b*) Describe any methods used to examine subgroups and interactions	8
		(*c*) Explain how missing data were addressed	8
		(*d*) *Cohort study*—If applicable, explain how loss to follow-up was addressed<br>*Case-control study*—If applicable, explain how matching of cases and controls was addressed<br>*Cross-sectional study*—If applicable, describe analytical methods taking account of sampling strategy	
		(*e*) Describe any sensitivity analyses	8
**Results**			
Participants	13*	(a) Report numbers of individuals at each stage of study—eg numbers potentially eligible, examined for eligibility, confirmed eligible, included in the study, completing follow-up, and analysed	9
		(b) Give reasons for non-participation at each stage	
		(c) Consider use of a flow diagram	
Descriptive data	14*	(a) Give characteristics of study participants (eg demographic, clinical, social) and information on exposures and potential confounders	9
		(b) Indicate number of participants with missing data for each variable of interest	
		(c) *Cohort study*—Summarise follow-up time (eg, average and total amount)	9
Outcome data	15*	*Cohort study*—Report numbers of outcome events or summary measures over time	10
		Case-control study—Report numbers in each exposure category, or summary measures of exposure	
		Cross-sectional study—Report numbers of outcome events or summary measures	
Main results	16	(*a*) Give unadjusted estimates and, if applicable, confounder-adjusted estimates and their precision (eg, 95% confidence interval). Make clear which confounders were adjusted for and why they were included	9, 10, 11
		(*b*) Report category boundaries when continuous variables were categorized	
		(*c*) If relevant, consider translating estimates of relative risk into absolute risk for a meaningful time period	

Continued on next page

For peer review only

Other analyses	17	Report other analyses done—eg analyses of subgroups and interactions, and sensitivity analyses	11
**Discussion**			
Key results	18	Summarise key results with reference to study objectives	12
Limitations	19	Discuss limitations of the study, taking into account sources of potential bias or imprecision. Discuss both direction and magnitude of any potential bias	14
Interpretation	20	Give a cautious overall interpretation of results considering objectives, limitations, multiplicity of analyses, results from similar studies, and other relevant evidence	15
Generalisability	21	Discuss the generalisability (external validity) of the study results	14, 15
**Other information**			
Funding	22	Give the source of funding and the role of the funders for the present study and, if applicable, for the original study on which the present article is based	16

*Give information separately for cases and controls in case-control studies and, if applicable, for exposed and unexposed groups in cohort and cross-sectional studies.

Note: An Explanation and Elaboration article discusses each checklist item and gives methodological background and published examples of transparent reporting. The STROBE checklist is best used in conjunction with this article (freely available on the Web sites of PLoS Medicine at http://www.plosmedicine.org/, Annals of Internal Medicine at http://www.annals.org/, and Epidemiology at http://www.epidem.com/). Information on the STROBE Initiative is available at www.strobe-statement.org.

For peer review only
